# GAB functions as a bioenergetic and signalling gatekeeper to control T cell inflammation

**DOI:** 10.1038/s42255-022-00638-1

**Published:** 2022-10-03

**Authors:** Siwen Kang, Lingling Liu, Tingting Wang, Matthew Cannon, Penghui Lin, Teresa W.-M. Fan, David A. Scott, Hsin-Jung Joyce Wu, Andrew N. Lane, Ruoning Wang

**Affiliations:** 1 Center for Childhood Cancer & Blood Diseases, Hematology/Oncology & BMT, Abigail Wexner Research Institute at Nationwide Children’s Hospital, Department of Pediatrics at The Ohio State University, Columbus, OH USA; 2 Center for Environmental and Systems Biochemistry, Department of Toxicology and Cancer Biology, Markey Cancer Center, University of Kentucky, Lexington, KY USA; 3 Cancer Metabolism Core, Sanford Burnham Prebys Medical Discovery Institute, La Jolla, CA USA; 4 Division of Rheumatology and Immunology, Department of Internal Medicine at The Ohio State University, Columbus, OH USA

**Keywords:** Autoimmunity, Extracellular signalling molecules, Metabolism, Inflammation

## Abstract

γ-Aminobutyrate (GAB), the biochemical form of (GABA) γ-aminobutyric acid, participates in shaping physiological processes, including the immune response. How GAB metabolism is controlled to mediate such functions remains elusive. Here we show that GAB is one of the most abundant metabolites in CD4^+^ T helper 17 (T_H_17) and induced T regulatory (iT_reg_) cells. GAB functions as a bioenergetic and signalling gatekeeper by reciprocally controlling pro-inflammatory T_H_17 cell and anti-inflammatory iT_reg_ cell differentiation through distinct mechanisms. 4-Aminobutyrate aminotransferase (ABAT) funnels GAB into the tricarboxylic acid (TCA) cycle to maximize carbon allocation in promoting T_H_17 cell differentiation. By contrast, the absence of ABAT activity in iT_reg_ cells enables GAB to be exported to the extracellular environment where it acts as an autocrine signalling metabolite that promotes iT_reg_ cell differentiation. Accordingly, ablation of ABAT activity in T cells protects against experimental autoimmune encephalomyelitis (EAE) progression. Conversely, ablation of GABA_A_ receptor in T cells worsens EAE. Our results suggest that the cell-autonomous control of GAB on CD4^+^ T cells is bimodal and consists of the sequential action of two processes, ABAT-dependent mitochondrial anaplerosis and the receptor-dependent signalling response, both of which are required for T cell-mediated inflammation.

## Abstract (web summary)

Kang et al. reveal that GAB, classically known as a neurotransmitter, can control pro-inflammatory T_H_17 cell and anti-inflammatory iT_reg_ cell differentiation in a cell-autonomous manner, thus modulating T cell-mediated inflammation.

## Main

**Fig. 1:**
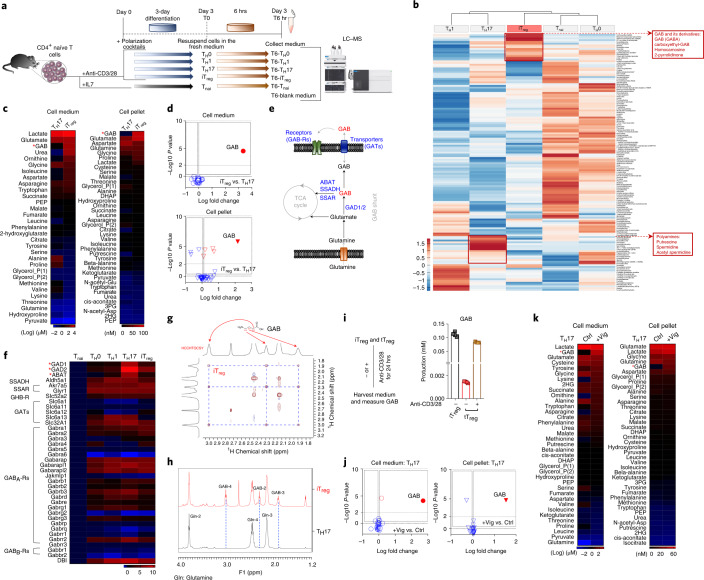
GAB is an abundant metabolite produced in T_H_17 and iT_reg_ cells. **a**, Experimental scheme of T cell extracellular metabolome profiles (LC–MS). **b**, Extracellular metabolites associated with amino acid metabolism in the indicated T cell subsets were profiled by LC–MS. The value for each metabolite represents *n* = 3 biologically independent samples. The heatmap represents the value of the relative amount (see colour scale). The complete metabolomic profile is provided as source data. **c**,**d**,**j**,**k**, Indicated metabolites were quantified by GC–MS. The value for each metabolite represents *n* = 3 biologically independent samples. Cysteinylglycine disulfide*, (2R)-2-amino-3-{[2-amino-2-(carboxymethylcarbamoyl)ethyl]disulfanyl}propanoic acid; alpha-ketoglutaramate*, 2-keto-glutaramate; 2-oxoarginine*, 5-[(diaminomethylidene)amino]-2-oxopentanoic acid; 2,3-dihydroxy-5-methylthio-4-pentenoate (DMTPA)*, (2R,3R,4E)-2,3-dihydroxy-5-(methylsulfanyl)pent-4-enoic acid. The heatmaps (**c**,**k**) represent the log value (medium) or the absolute value (pellet) of the indicated metabolite quantity (see colour scale). The complete data are provided as source data. The volcano plots (**d**,**j**) show changes in metabolites in the cell medium and cell pellet. Heatmaps and volcano plots are representative of *n* = 3 independent biological samples from *n* = 2 independent biological experiments. **e**, Schematic of the pathway of GABA metabolism. **f**, RNA was isolated from the indicated T cell subsets (*n* = 3 biologically independent samples) and used for qPCR analyses of the indicated metabolic genes. mRNA levels of T_nai_ cells were set to 1. The heatmap represents the log value of the relative mRNA expression level (see colour scale). Values and s.d. are provided as source data. **g**,**h**, GAB in the indicated groups was determined and quantified by NMR (*n* = 3 biologically independent samples). **i**, As illustrated by the experimental scheme (left), GAB production in iT_reg_ and tT_reg_ cells was quantified by a GAB bioassay kit (*n* = 4 biologically independent samples). Statistical analysis was performed by R (**b**) or unpaired two-tailed Student’s *t-*test (**c,d**,**i**–**k**). GAD, glutamate decarboxylase; SSADH, succinic semialdehyde dehydrogenase; SSAR, succinic semialdehyde reductase; GHB-R, γ-hydroxybutyrate receptor; GATs, GABA transporters; VGAT, vesicular GABA transporter; GABA_A_-R, GABA type A receptor; GABA_B_-R, GABA type B receptors; DBI, diazepam binding inhibitor. Source data

Mounting a robust and effective adaptive immune response in vertebrates is metabolically costly and requires proper allocation of essential yet limited energy and carbon resources. Metabolism must be tightly controlled at the cellular level to coordinate rapid expansion followed by a fine-tuned differentiation process in T cells. Beyond acting as bioenergetic substrates and biosynthetic precursors, metabolites can directly control cellular signalling responses through influencing DNA, RNA and protein modifications, signalling receptors’ activities and the production of reactive oxygen species^1–5^. As such, metabolism is fundamental to fine-tuning carbon and nitrogen allocation and optimizing immune response, which is at the centre of many diseases. Previous studies have used systemic approaches to comprehensively characterize the transcriptome, the abundance of intracellular metabolites and the overall catabolic activities of T cells at the different stages during the T cell life cycle^6,7^. These studies have generated critical temporal snapshots of the metabolic landscapes, which help establish a conceptual foundation for understanding T cell metabolic reprogramming. However, most of these studies have centred mainly on intracellular metabolites and activities of the central carbon metabolism. The overall metabolic landscape of T cells can also be delineated by monitoring the metabolites consumed from and secreted into the growth medium. The extracellular metabolome represents the ultimate outcome of metabolic input, processing and output. Extracellular metabolome profiling (also called metabolic footprinting) has been applied as a standard technique to optimize microbial bioprocesses by analysing substrates consumed from and metabolites secreted into a microorganism’s culture medium^8,9^. Here, we took a similar approach (Fig. 1a) to compare the extracellular metabolome profiles of naive T (T_nai_) cells and different subsets of effector T (T_eff_) cells, including T helper (T_H_0, T_H_1, T_H_17) cells and induced regulator T (iT_reg_) cells.

## Results

### GAB is an abundant metabolite produced by effector T cells

**Extended Data Fig. 1:**
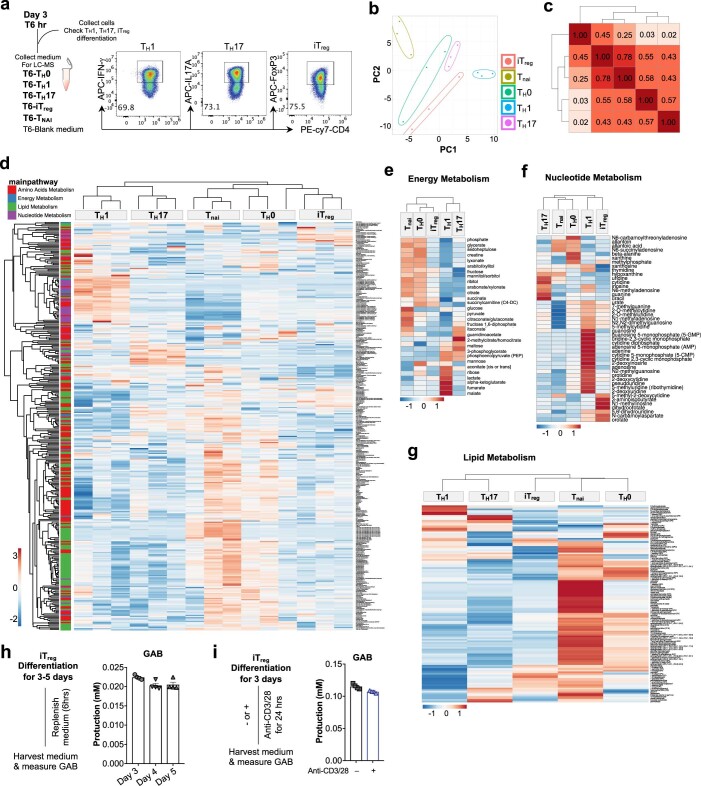
Distinctive extracellular metabolome profiles characterize T cell subsets. **a**, Cytokine production of indicating T cell subsets was determined by flow cytometer. **b**, Principal component analysis (PCA) for the correlations among each subset. **c**, Pairwise comparison of the statistical analysis, the numbers reflect the correlation R values. **d**, Extracellular metabolites in indicated T cell subsets were profiled by LC-MS. The hierarchical clustering heatmap represents the value of the relative amount (see color scale). **e-g**, Extracellular metabolites associated with energy metabolism (**e**), nucleotide metabolism (**f**), and lipid metabolism (**g**) in indicated T cell subsets were profiled by LC-MS. The value for each metabolite represents the average of triplicates. The complete metabolomic profile is provided in ***Source Data file 5***. Statistical analysis was performed by R Programming Language (**b–g**). **h, i**, As illustrated by the experimental scheme (left), GAB production of iTreg cells in indicated conditions was quantified by a GAB bioassay kit (*n* = 4 biologically independent samples). Source data

The control (blank) medium and the spent medium of different subsets of T_eff_ cells (Extended Data Fig. 1a) were profiled on a semi-quantitative untargeted global metabolomics platform based on liquid chromatography–mass spectrometry (LC–MS), with broad coverage of up to 1,000–2,500 compounds, including amino acids, energy metabolites, nucleotides and lipids. Using this approach, we have classified metabolites as having changes in production or consumption according to whether the fold change compared with control was positive or negative, respectively. Hierarchical clustering analysis, the pairwise comparison and the principal-component analysis revealed that T cell subsets were characterized by distinct extracellular metabolome profiles (Fig. 1b and Extended Data Fig. 1b–g). Consistent with the role of central carbon metabolism in supporting cell growth, the hyper-proliferative T_eff_ groups consumed more carbohydrates and produced more lactate than the T_nai_ group (Extended Data Fig. 1e). Additionally, the T_H_17 group was characterized by the highest production of polyamines (Fig. 1b), in line with the recent finding of a critical role for polyamine in determining T_H_17 differentiation^10–12^. Intriguingly, iT_reg_ cells produced high levels of γ-aminobutyrate (GAB) and its derivatives (Fig. 1b). Next, we applied gas chromatography–MS (GC–MS)-based targeted metabolomics and nuclear magnetic resonance (NMR) to validate and quantify intracellular and extracellular GAB production. We confirmed that iT_reg_ cells produced much higher levels of GAB than T_H_17 cells (Fig. 1c–h). Unexpectedly, GAB was the most abundant intracellular metabolite and among the top three extracellular metabolites in iT_reg_ cells (Fig. 1c,d). However, neither prolonged culture nor restimulation would significantly changed GAB excretion (Extended Data Fig. 1h,i). Following activation, thymus-derived T_reg_ (tT_reg_) cells could also excrete a comparable amount of GAB to the medium as iT_reg_ cells (Fig. 1i). Notably, the intracellular level of GAB was even higher than that of glutamate (Glu) in iT_reg_ cells, which is one of the most abundant intracellular metabolites in various organisms^13^. GAB is produced by catabolizing glutamine (Gln) through the (GABA) γ-aminobutyric acid shunt and elicits GABAergic response through GABA receptors (GABA-Rs) in neurons. To better understand the molecular nature that determines GAB production and function in T cells, we examined the expression of a panel of GABA-related metabolic and receptor genes by qPCR (Fig. 1e,f). Consistent with the previous findings on the GABA-R expression profile in immune cells^14^. T_eff_ cells expressed a selected group of GABA-R subunits. However, only iT_reg_ and T_H_17 cells expressed high levels of glutamate decarboxylase (GAD), the enzyme that catalyzes the decarboxylation of Glu to GAB. Unexpectedly, the T_H_17 group exhibits a higher level of GAD than the iT_reg_ group and was the only group that expressed a high level of the GAB-catabolizing enzyme 4-aminobutyrate aminotransferase (ABAT), indicating increased GAB catabolism in T_H_17 cells but not in iT_reg_ cells (Fig. 1f**)**. Collectively, these findings suggest that extracellular metabolome profiling is a robust approach to revealing T cell metabolic characteristics in vitro. Using this approach, we have found that GAB is an abundant metabolite produced by T cells.

### T cells use both Gln and Arg to produce GAB

**Fig. 2:**
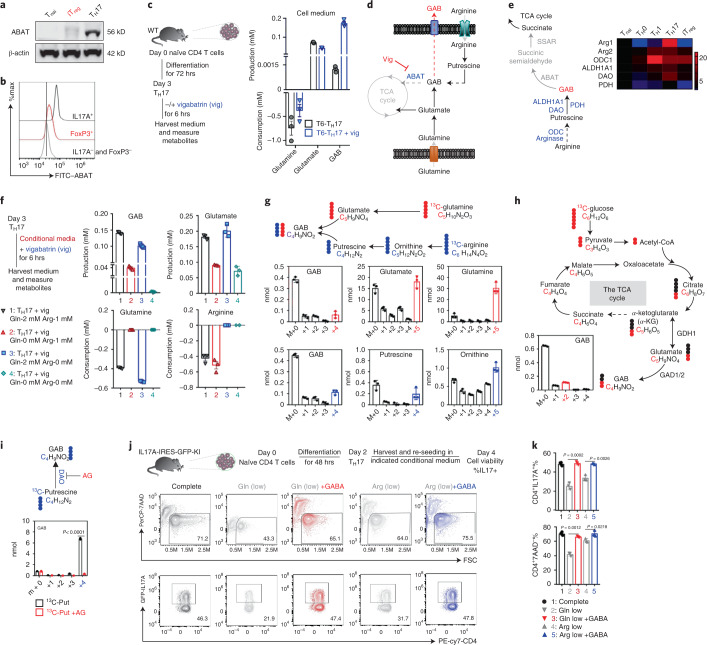
Gln and Arg are the main carbon sources for GAB biosynthesis in T_eff_ cells. **a,b**, ABAT protein levels were determined by IB (ABAT molecular weight, 56 kDa; full-scan images are provided as source data) (**a**) and cytometry (**b**). **c**, As illustrated by the experimental scheme (left), the indicated metabolites in T_H_17 cells were quantified by GC–MS (right); data are representative of *n* = 3 biologically independent samples. **d**, Scheme of the GAB metabolic pathways and a pharmacological inhibitor Vig of ABAT. **e**, Schematic diagram of GAB biosynthesis from Arg (left), with the expression of relevant genes determined by qPCR detection (right). mRNA levels for T_nai_ cells were set to 1. The heatmap represents the relative mRNA expression level (see colour scale). Values and s.d. are provided as source data. **f**. As illustrated by the experimental scheme (left), the indicated metabolites in T_H_17 cells were quantified by YSI (Gln and Glu) or by bioassay kits (Arg and GAB) (*n* = 3 biologically independent samples). **g**, Diagram of conversion of [^13^C_5_]Glu and [^13^C_6_]Arg to downstream metabolites (top). Indicated metabolites in T_H_17 cells were quantified by GC–MS (*n* = 3 biologically independent samples) (bottom). Numbers along the *x* axis represent those of ^13^C atoms in the given metabolites. **h**, Diagram of the conversion of [^13^C_6_]Glc to downstream metabolites. Indicated metabolites in T_H_17 cells were quantified by GC–MS (*n* = 3 biologically independent samples). Black dot, ^12^C; red dot, ^13^C derived from [^13^C_6_]Glc. **i**, Diagram of the conversion of [^13^C_4_]Put to GAB. Indicated metabolites in T_H_17 cells were quantified by GC–MS (*n* = 3 biologically independent samples). Unpaired two-tailed Student’s *t-*test. **j**, As illustrated by the experimental scheme (top), cytokine expression and cell viability were determined by flow cytometry (bottom). All experiments with bicuculline (5 μM); complete medium, 2 mM Gln + 0.1 mM Arg; Gln (low), 10 μM Gln + 0.1 mM Arg; Gln (low plus GABA), 10 μM Gln + 0.1 mM Arg + 1 mM GABA; Arg (low), 2 mM Gln + 10 μM Arg; Arg (low plus GABA) 2 mM Gln + 10 μM Arg + 1 mM GABA. **k**, Statistical analysis for **j**; data are representative of *n* = 3 biologically independent samples. Significance was calculated by one-way ANOVA with Tukey’s multiple-comparisons test. Gln, glutamine; Glu, glutamate; Arg, arginine; ODC, ornithine decarboxylase; DAO, diamine oxidase; PDH, pyrroline dehydrogenase; M, million. Source data

Given the higher expression of GAD and ABAT in T_H_17 cells relative to iT_reg_ cells, we reasoned that both iT_reg_ cells and T_H_17 cells could produce GAB. However, the fate of GAB depends on ABAT, that is, GAB is diverted into the tricarboxylic acid (TCA) cycle in the presence of ABAT in T_H_17 cells instead of being exported into the extracellular compartment in the absence of ABAT as in iT_reg_ cells. To test this idea, we cultured T_H_17 cells with or without the potent ABAT inhibitor vigabatrin (Vig)^15,16^, for 6 h and then measured the levels of a panel of metabolites. Inhibiting ABAT activity by Vig led to the accumulation of intracellular GAB and GAB release into the medium (Fig. 1k,j). Notably, inhibiting ABAT activity rendered GAB one of the most abundant metabolites in the medium and cell pellet (Fig. 1k,j). Moreover, we have validated that ABAT was expressed in T_H_17 cells but not in iT_reg_ cells using immunoblot (IB) analysis and intracellular staining (Fig. 2a,b). Interestingly, inhibiting ABAT activity reduced Gln consumption without changing Glu levels significantly but increased GAB levels over 100-fold (Fig. 2c,d). The reciprocal changes in Gln consumption versus GAB production raise the possibility of a Gln-independent GAB production route in T_H_17 cells. Gln catabolism via the GABA shunt is the canonical GAB biosynthesis pathway^17^. Alternatively, GAB could be formed from putrescine (Put), a metabolite mainly derived from arginine (Arg) (Fig. 2d)^18,19^. Indeed, the metabolic genes involved in converting Arg into GAB were highly expressed in T_H_17 cells (Fig. 2e). To determine to what extent Gln and Arg contribute to GAB biosynthesis, we cultured T_H_17 cells with Vig in the presence or absence of Gln, Arg or both. Then, we collected spent medium to measure the levels of various metabolites. While removing Gln or Arg reduced GAB production, the removal of both completely blocked GAB production (Fig. 2f). Next, we supplied [^13^C_6_]Arg, [^13^C_5_]Gln, [^13^C_6_]glucose (Glc) or [^13^C_4_]Put as metabolic tracers in the culture medium and followed ^13^C incorporation into individual metabolites by GC–MS. The presence of the ^13^C_4_ isotopologue of GAB and the corresponding ^13^C_4_ or ^3^C_5_ isotopologues of upstream metabolites further confirmed that Gln and Arg are carbon donors of GAB (Fig. 2g). However, only the ^13^C_2_ isotopologue of GAB was detected in samples with [^13^C_6_]Glc, suggesting that Glc can support Glu (and GAB) synthesis through the TCA cycle (Fig. 2h). Finally, we showed that Put can be converted to GAB via a diamine oxidase (DAO)-dependent reaction as its inhibitor aminoguanidine (AG) completely blocked the production of [^13^C_4_]GAB from [^13^C_4_]Put (Fig. 2i). In addition to a general requirement of both amino acids for protein synthesis, we envisioned that Gln and Arg might also support T_H_17 function and survival through supporting GAB biosynthesis. To test this idea, we cultured T_H_17 cells in Gln/Arg-replete medium or suboptimal medium (with low levels of Gln/Arg) in the absence or presence of high levels of GAB. Supporting our hypothesis, reducing the amount of either amino acid led to defects in the maintenance of viability and interleukin (IL)-17^+^ populations. Notably, GAB supplementation could correct both defects (Fig. 2j,k). We, therefore, conclude that T_H_17 cells can use both Gln-derived and Arg-derived carbon to synthesize GAB and support cell viability and function.

### ABAT confers GAB-dependent anaplerosis on T_H_17 cells

**Fig. 3:**
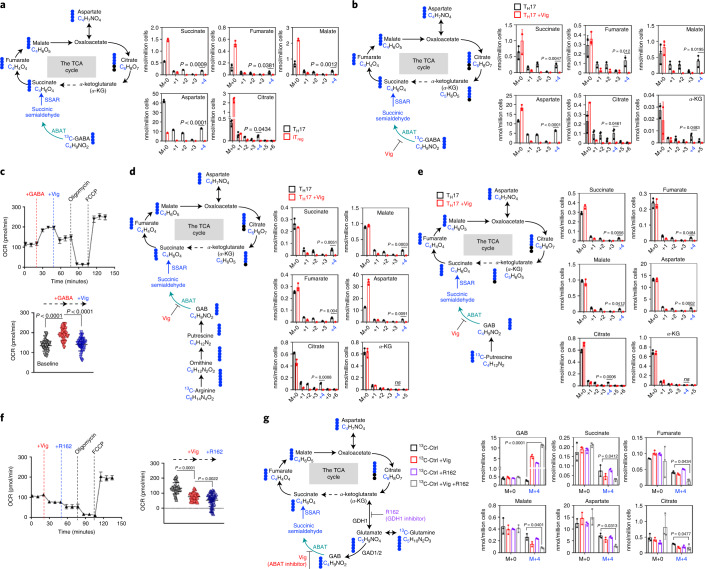
ABAT enables diversion of GAB into the TCA cycle in T cells. **a**,**b**,**c**,**d**,**e**,**g**,Diagrams of the conversion of [^13^C_4_]GABA (**a**,**b**, left), [^13^C_6_]Arg (**d**, left), [^13^C_4_]Put (**e**, left) and [^13^C_5_]Gln (**g**, left) to downstream metabolites. Indicated metabolites in T_H_17 cells were quantified by GC–MS (*n* = 3 biologically independent samples) (right). Black dot, ^12^C; blue dot, ^13^C derived from the indicated tracers. Numbers along the *x* axis represent those of ^13^C atoms in the given metabolites. Significance was calculated by unpaired two-tailed Student’s *t-*test (**a, b, d, e** and **g**); NS, no significant differences. **c,f**, OCR of T_H_17 cells with the indicated treatments was determined by Seahorse. Data are representative of *n* = 16 independent biological samples from *n* = 3 independent biological experiments. Two-way ANOVA with Sidak’s multiple-comparisons test (**c**) and (**f**). α-KG, α-ketoglutarate; GDH, glutamate dehydrogenase.

Next, we reasoned that the expression of ABAT may render T_H_17 cells capable of diverting GAB into the TCA cycle in a way that maximizes carbon allocation and oxidative phosphorylation (OXPHOS) in mitochondria. To test this idea, we added [^13^C_4_]GABA as a metabolic tracer into the culture medium and followed ^13^C incorporation into intermediate metabolites of the TCA cycle in iT_reg_ cells and T_H_17 cells with or without Vig treatment (Fig. 3a,b). In line with the expression of ABAT in T_H_17 cells but not in iT_reg_ cells, T_H_17 cells exhibited much higher levels of the ^13^C_4_ isotopologue of succinate and its downstream metabolites in the TCA cycle than iT_reg_ cells (Fig. 3a). Inhibiting ABAT activity by Vig completely abolished the ^13^C_4_ isotopologue of succinate and its downstream metabolites in T_H_17 cells, supporting the idea that GAB is diverted to the TCA cycle via an ABAT-dependent reaction (Fig. 3b). Next, we sought to determine the temporal change in respiration following a sequential supplementation of GABA, Vig, oligomycin or carbonyl cyanide-*p*-trifluoromethoxyphenylhydrazone (FCCP) into the T_H_17 cell culture medium. Indeed, GABA supplementation enhanced oxygen consumption in an ABAT-dependent manner, while ATPase inhibitor oligomycin suppressed and FCCP maximized oxygen consumption as expected (Fig. 3c). We and others have recently shown that Arg-dependent polyamine biosynthesis is required to support T cell proliferation and T_H_17 cell differentiation^10–12^. We reasoned that ABAT expression in T_H_17 cells might allow Arg-derived carbons to be diverted into the TCA cycle through Put and GAB. Supporting this idea, [^13^C_6_]Arg-derived and [^13^C_4_]Put-derived ^13^C were incorporated into the ^13^C_4_ isotopologue of succinate and its downstream metabolites in an ABAT-dependent manner in T_H_17 cells (Fig. 3d, e). Finally, we sought to determine whether Gln-derived carbons could enter the TCA cycle via ABAT. Gln is a major carbon donor known to drive the TCA cycle and OXPHOS via glutamine transaminase and glutamate dehydrogenase (GDH) in T_eff_ cells^20–22^. We found that a sequential supplementation with Vig and the GDH inhibitor R162 (ref. ^23^) suppressed oxygen consumption additively (Fig. 3f). Similarly, combining Vig and R162 suppressed [^13^C_5_]Gln-derived ^13^C incorporation into the TCA cycle metabolites more profoundly than single-agent treatment (Fig. 3g). Collectively, we have identified GAB as a conditional anaplerotic substrate in T cells, and its catabolism via the TCA cycle depends on the expression of ABAT.

### GAB metabolism controls proliferation and differentiation

**Fig. 4:**
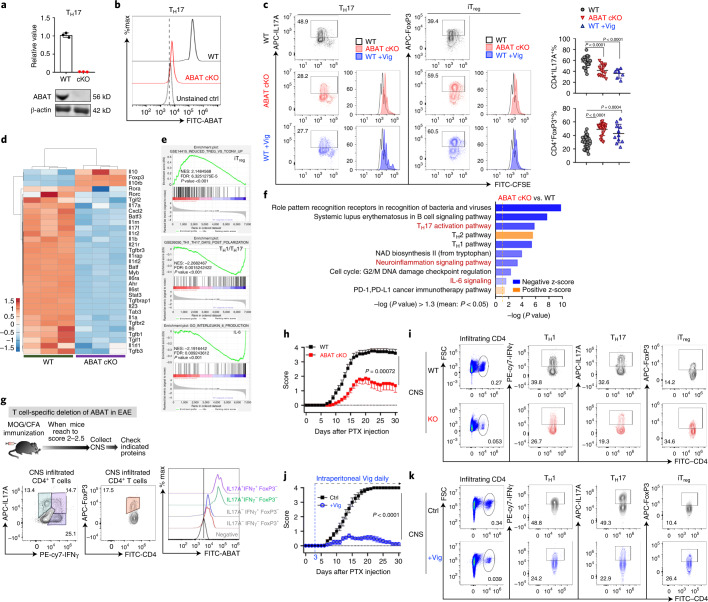
Genetic ablation or pharmacological inhibition of ABAT suppresses T_H_17 but enhances iT_reg_ cell differentiation. **a**,**b**, ABAT mRNA and protein levels were determined by qPCR and IB analysis (**a**, full-scan images are provided in as source data) or by flow cytometry (**b**); unpaired two-tailed Student’s *t-*test for **a** (*n* = 3 biologically independent samples). **c**, Expression of the indicated cytokines and CFSE dilution in the indicated groups determined by flow cytometry (WT, *n* = 5 independent biological samples from *n* = 5 independent biological experiments; *Abat* cKO, *n* = 5 independent biological samples from *n* = 3 independent biological experiments; WT plus Vig, *n* = 3 independent biological samples from *n* = 3 independent biological experiments). Two-way ANOVA with Sidak’s multiple-comparisons test. **d**–**f**, Hierarchical clustering analysis, gene set enrichment analysis (GSEA) and Ingenuity Pathway Analysis (IPA) of a list of inflammatory genes performed by using RNA-seq data of CD4^+^ T cells that were cultured under T_H_0 culture conditions and collected at 36 h after activation (*n* = 3 independent biological samples). In **d**, heatmaps represent the log-transformed value of the quantity (see colour scale). In **f**, the orange dotted line along the *x* axis indicates the cut-off value (*P* = 0.05). The complete data are provided as source data. **g**, As illustrated by the experimental scheme (top), the expression of the indicated proteins in CNS-infiltrating CD4^+^ T cells isolated from EAE animals was determined by flow cytometry (*n* = 3 independent biological samples from *n* = 2 independent biological experiments). **h**,**j**, EAE clinical scores in the indicated groups evaluated daily from mice of the indicated genotypes (*n* = 6 independent biological samples from *n* = 2 independent biological experiments in **h** and *n* = 5 independent biological samples from *n* = 3 independent biological experiments in **j**); significance was calculated by unpaired two-tailed Student’s *t-*test. **i**,**k**, The expression of the indicated markers in CNS-infiltrating T cells determined by flow cytometry (*n* = 5 independent biological samples in **i** and *n* = 3 independent biological samples in **k**). PTX, paclitaxel; NES, normalized enrichment score; FDR, false discovery rate. Source data

**Extended Data Fig. 2:**
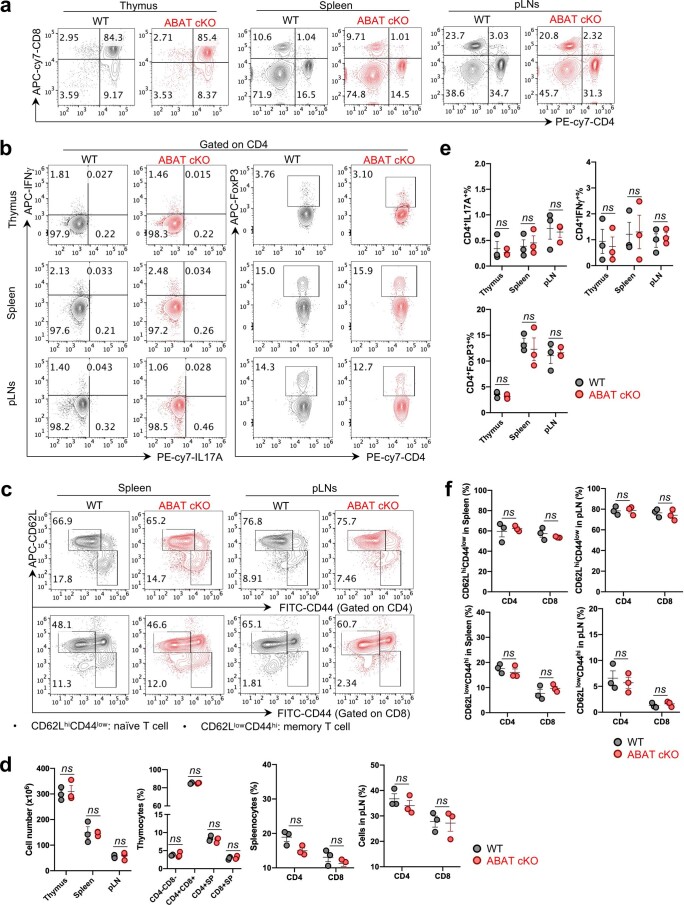
ABAT is dispensable for normal T cell development after the double-positive stage. **a–f**, Distribution of CD4^+^ and CD8^+^ T cell (**a, d**), indicated intracellular proteins (**b, e**) and surface markers (**c, f**) were determined by flow cytometer. Data are shown as mean ± SEM, *n* = 3 biologically independent samples, significance was calculated by unpaired Two-tail Student’s t-test. *ns*, no significant differences.

**Extended Data Fig. 3:**
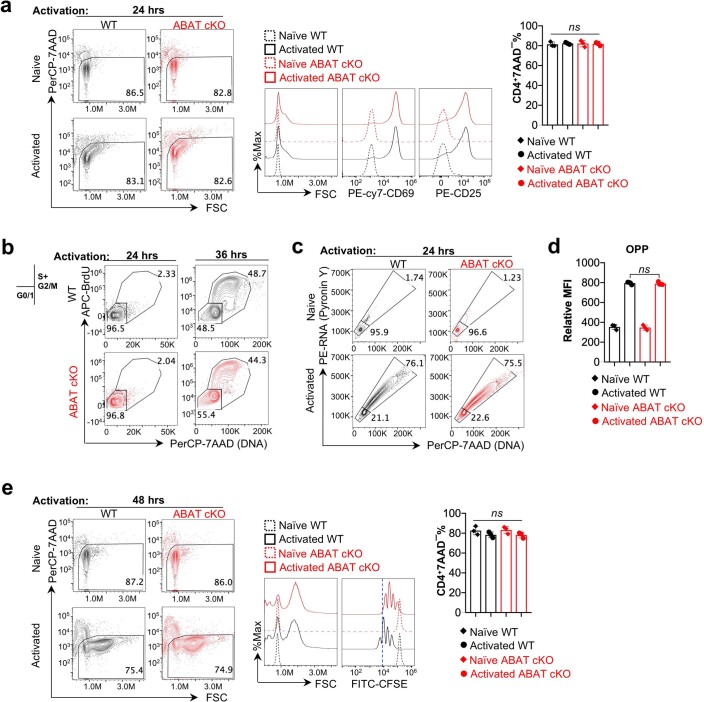
ABAT is dispensable for T cell activation. **a**, Cell viability, size and activation markers (**a**), cell cycle profile (**b**), DNA/RNA contents (**c**), protein synthesis activity (**d**), and CFSE dilution and cell viability (**e**) were determined by flow cytometry. Data are shown as mean ± SEM, *n* = 3 biologically independent samples, significance was calculated by Two-way ANOVA with Sidak’s multiple comparisons test. *ns*, no significant differences. OPP: o-propargyl-puromycin, MFI: median fluorescence intensity.

**Extended Data Fig. 4:**
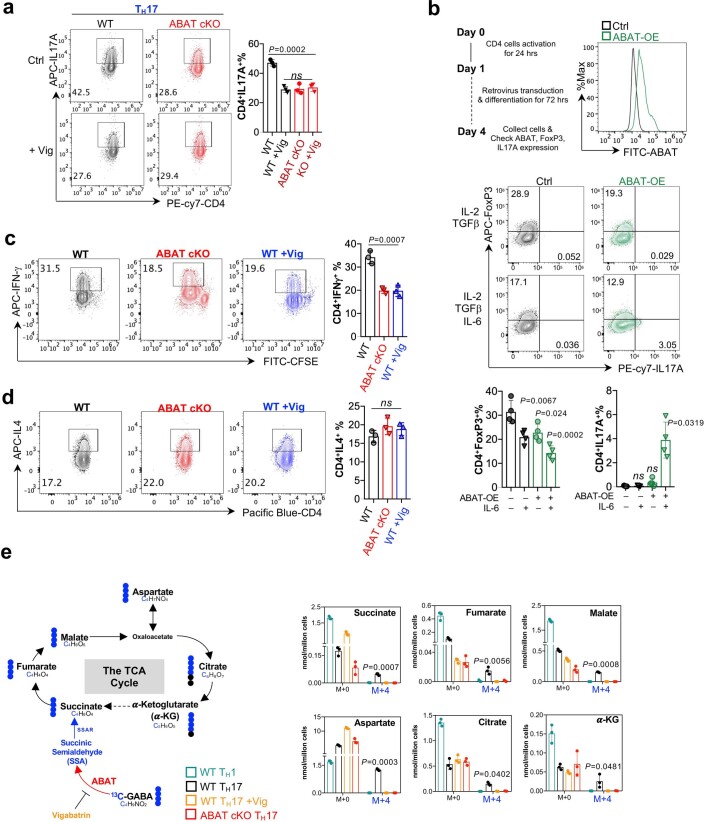
ABAT regulates T cell differentiation *in vitro*. **a–d**, Expression of indicated cytokines in indicated groups was determined by flow cytometry: **a, c, d**, data represents of *n* = 3 biologically independent samples, significance was calculated by one-way ANOVA with Tukey’s multiple comparisons test. *ns*, no significant differences. **b**, data represents of *n* = 4 biologically independent samples, unpaired Two-tail Student’s t-test. ns, no significant differences. **e**, Diagram of converting [^13^C_4_]-GABA (left) to downstream metabolites. Indicated metabolites were quantified by GC-MS (*n* = 3 biologically independent samples). Black dot: ^12^C; Blue dot: ^13^C derived from the indicated tracers. Numbers in the X-axis represent those of ^13^C atoms in given metabolites. Significance was calculated by unpaired Two-tail Student’s t-test. ABAT-OE: ABAT overexpression, α-KG: α-Ketoglutarate, M: mass spectrum.

To further delineate the role of ABAT in T cells, we generated a T cell-specific *Abat*-knockout strain (*Abat* cKO) by crossing the *Abat*^*fl*^ strain with the Cd4-Cre strain. qPCR, IB and intracellular staining analyses validated the deletion of ABAT (Fig. 4a, b). ABAT deletion did not result in T cell development defects in the thymus, the spleen or lymph nodes (Extended Data Fig. 2a–f). In addition, ABAT deletion did not affect cell viability, the expression of cell surface activation markers, the cell cycle progression from G0/G1 to the S phase, RNA, DNA or protein contents, cell size, or viability 24 h after activation in vitro (Extended Data Fig. 3a–d). However, ABAT deletion moderately suppressed overall T cell proliferation after activation in vitro (Fig. 4c and Extended Data Fig. 3e). Remarkably, both genetic and pharmacological ablation of ABAT activity inhibited pro-inflammatory T_H_17 cell differentiation while enhancing anti-inflammatory iT_reg_ cell differentiation in vitro (Fig. 4c). Supporting these findings, the RNA-seq analysis of wild-type (WT) and *Abat* cKO T cells activated under the T_H_0 condition revealed enriched gene signatures associated with inflammation and T cell differentiation (Fig. 4d–f). Notably, the ABAT inhibitor (Vig) did not potentiate the effect of genetic deletion in suppressing T_H_17 cell differentiation, suggesting that Vig is a specific inhibitor of ABAT (Extended Data Fig. 4a). Moreover, overexpressing ABAT (ABAT-OE) suppressed iT_reg_ differentiation and could synergize with IL-6 to increase the percentage of IL-17^+^ cells under the iT_reg_-polarizing condition in vitro (Extended Data Fig. 4b). Next, we examined the effect of ABAT inhibition on T_H_1 and T_H_2 differentiation. Genetic and pharmacological ablation of ABAT activity inhibited T_H_1 cell differentiation without significantly changing T_H_2 cell differentiation significantly in vitro (Extended Data Fig. 4c, d). Finally, we asked whether T_H_1 cells could divert GAB into the TCA cycle as T_H_17 cells did. We applied [^13^C_4_]GABA as a metabolic tracer and followed ^13^C incorporation into intermediate metabolites of the TCA cycle in T_H_1 and T_H_17 cells. Only T_H_17 cells exhibited high levels of the ^13^C_4_ isotopologue of succinate and its downstream metabolites in the TCA cycle (Extended Data Fig. 4e**)**. Notably, genetic and pharmacological ablation of ABAT activity completely abolished the ^13^C_4_ isotopologues of metabolites in T_H_17 cells (Extended Data Fig. 4e).

**Extended Data Fig. 5:**
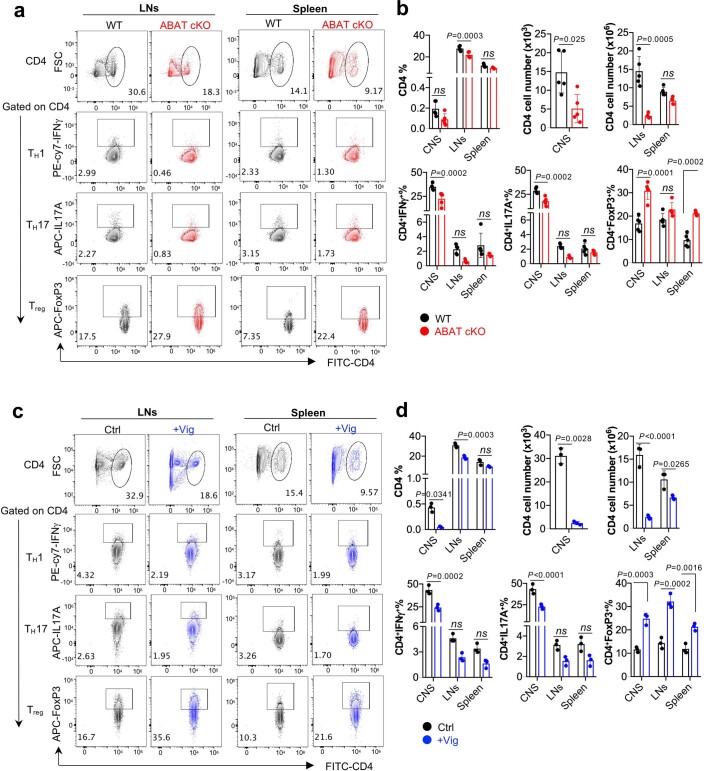
Genetic ablation or pharmacological inhibition of ABAT reduces T cell inflammation in EAE. **a–d**, T cells were isolated from indicated sites in experimental animals described in Fig. 4h (**a, b**) and Fig. 4j (**c, d**). The expression of indicated proteins was determined by flow cytometry. Statistical analysis (*n* = 3 biologically independent samples) was calculated by unpaired Two-tail Student’s t-test. Data are shown as mean ± SEM, *ns*, no significant differences. Vig: vigabatrin, EAE: experimental autoimmune encephalomyelitis, CNS: central nervous system, LNs: lymph nodes.

**Extended Data Fig. 6:**
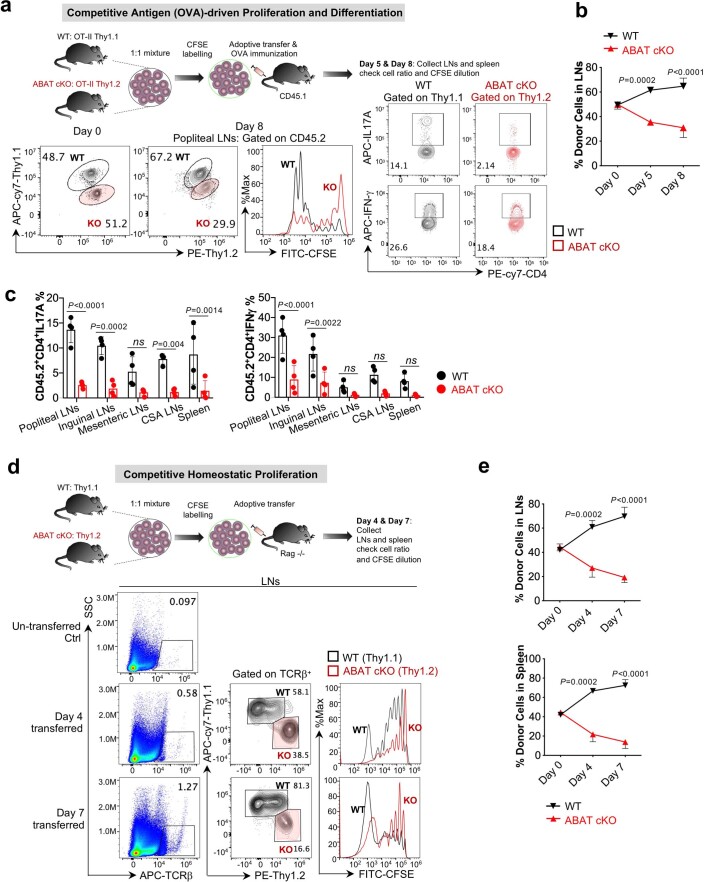
Inhibition of ABAT suppresses T cell proliferation and differentiation *in vivo*. **a–c**, As illustrated by the experimental diagram of competitive antigen (OVA)-specific proliferation (**a**, top), the donor cell ratio (**a**, bottom and **b**), CFSE dilution (**a**, bottom), and indicated protein levels (**a**, bottom and **c**), were determined by flow cytometry (*n* = 3 biologically independent samples). **b**, **c**, Data are shown as mean ± SEM, significance was calculated by unpaired Two-tail Student’s t-test. *ns*, no significant differences. **d–e**, As illustrated by the experimental diagram of competitive homeostatic proliferation (**d**, top). The donor cell ratio (**d**, bottom, and **e**) and CFSE dilution (**d**, bottom) were determined by flow cytometry (*n* = 3 biologically independent samples). **e**, Significance was calculated by unpaired Two-tail Student’s t-test. CSA: cervical, submandibular and axilla.

The expansion and balance between pro-inflammatory CD4^+^ T_eff_ cells and anti-inflammatory CD4^+^ T_reg_ cells determine the pathogenic development of experimental autoimmune encephalomyelitis (EAE), a mouse model of multiple sclerosis (MS), which is an inflammatory demyelinating disease of the central nervous system (CNS). Consistent with the expression profile of ABAT in vitro, the IL-17^+^CD4^+^ T cell group expressed the highest level of ABAT among all the CD4^+^ T subsets with infiltration into the CNS in animals with EAE (Fig. 4g). Notably, the genetic deletion of *Abat* in T cells or the systemic delivery of Vig conferred significant protection against EAE pathogenic progression, associated with more infiltrated FoxP3^+^CD4^+^ T cells and fewer infiltrated inflammatory CD4^+^ T cells, reciprocally (Fig. 4h,i and Extended Data Fig. 5a,b). However, Vig treatment resulted in better protection against EAE and a broader impact on periphery CD4^+^ T cells in the periphery than T cell-specific deletion of *Abat*, indicating that the systemic inhibition of ABAT might affect inflammation through both T cell-intrinsic and T cell-extrinsic mechanisms (Fig. 4j,k, and Extended Data Fig. 5c,d). We also used a competitive antigen-specific, T cell receptor (TCR)-dependent proliferation assay (OT-II) and a competitive homeostatic proliferation assay to assess T cell proliferation and differentiation in vivo. Notably, the ratio between WT and *Abat* cKO CD4^+^ T cells, CFSE dilution patterns and the percentage of IL-17^+^CD4^+^ or interferon-γ (IFNγ^+^)CD4^+^ T cells in various tissues suggested that the loss of ABAT dampens T cell proliferation and T_H_1 and T_H_17 differentiation in vivo (Extended Data Fig. 6a–e). Collectively, our results indicate that ABAT status determines the fate of intracellular GAB and, hence, pro-inflammatory T_H_17 and anti-inflammatory iT_reg_ cell differentiation in vitro and in vivo.

### GAB regulates T cell differentiation through the GABA_A_ receptor

**Fig. 5:**
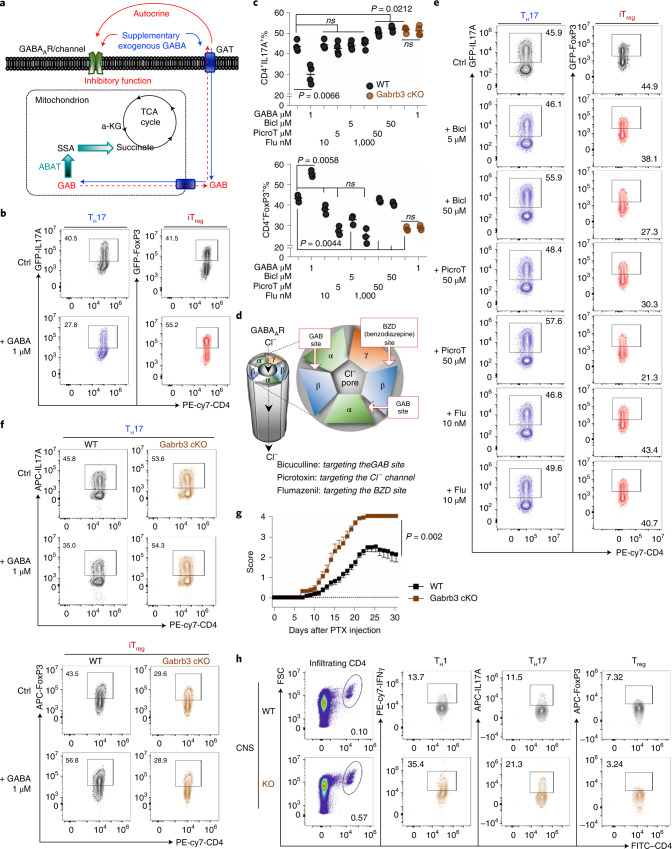
Receptor-mediated GAB autocrine signalling response reciprocally suppresses T_H_17 and enhances iT_reg_ differentiation. **a**,**d**, Schematic diagram of GAB metabolism and the GABA_A_-R-mediated autocrine signalling response in T cells (**a**) and schematic diagram of the binding sites for GABA_A_-R antagonists (**d**). **b**,**e**,**f**, Cytokine expression of the indicated groups determined by flow cytometry. **c**, Combination statistical analysis of **b**, **e** and **f;** data are shown as mean ± s.e.m. (*n* = 4 independent biological samples). Significance was calculated by two-way ANOVA with Sidak’s multiple-comparisons test. **g**, EAE clinical scores in the indicated groups evaluated daily (*n* = 5 independent biological samples from *n* = 3 independent biological experiments); significance was calculated by unpaired two-tailed Student’s *t-*test. **h**, Expression of the indicated markers in CNS-infiltrating T cells were determined by flow cytometry (*n* = 3 independent biological samples). Bicl, bicuculline; PicroT, picrotoxin; Flu, flumazenil; PTX, paclitaxel.

**Extended Data Fig. 7:**
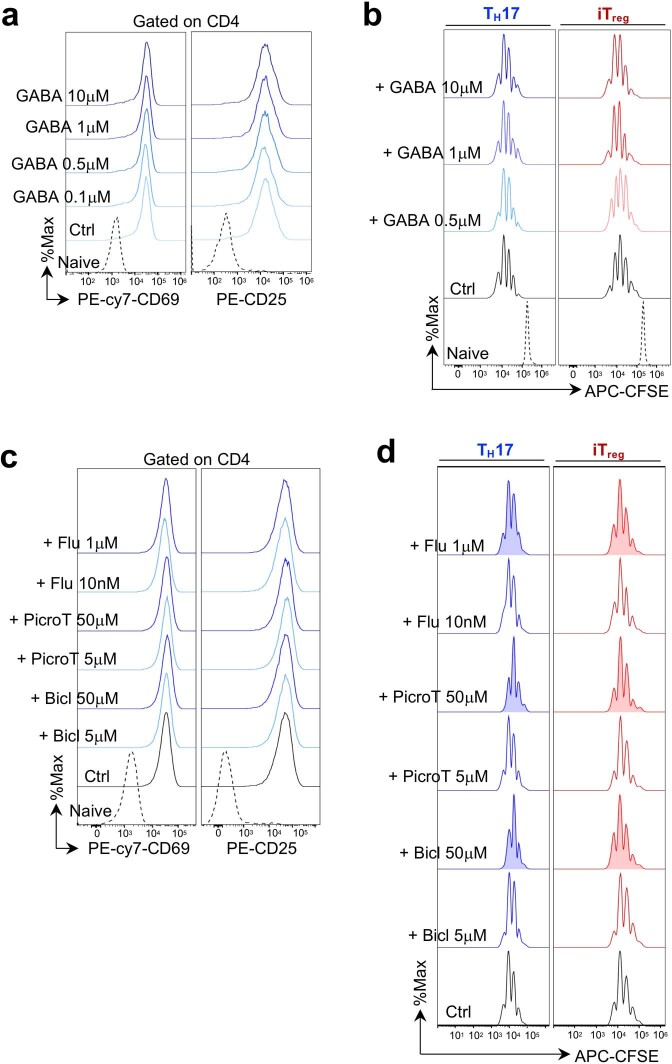
Modulating GABA receptor-mediated response does not affect activation and proliferation significantly during T_H_17 and iT_reg_ differentiation. **a–d**, Cell activation markers (**a, c**), and CFSE dilution (**b, d**) were determined by flow cytometry (*n* = 3 biologically independent experiments). Bicl: bicuculline, PicroT: picrotoxin, Flu: flumazenil.

**Extended Data Fig. 8:**
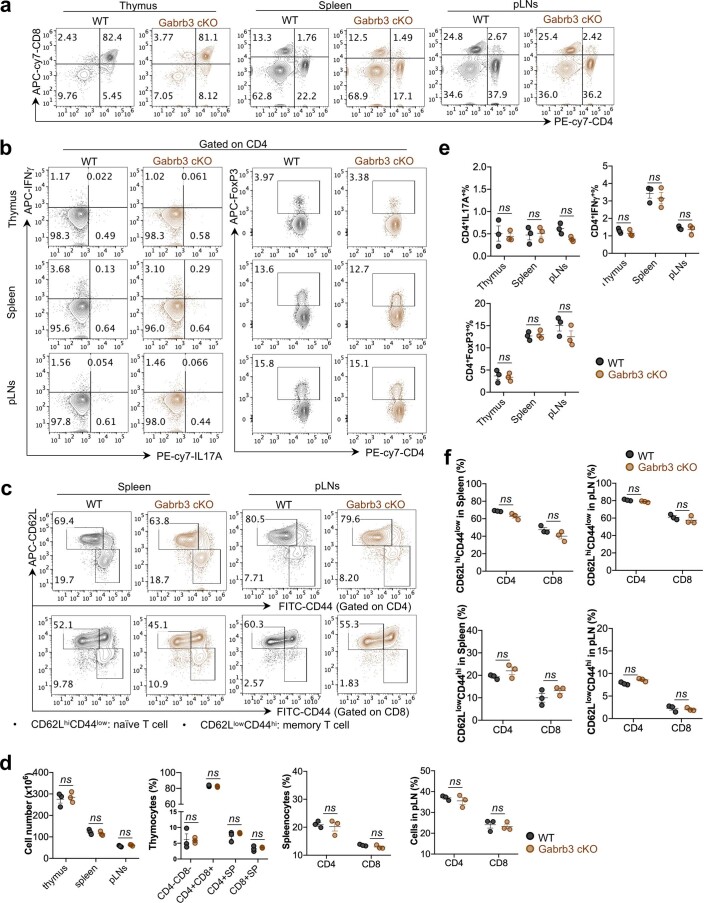
GABA receptor is dispensable for normal T cell development after the double-positive stage. **a–f**, Distribution of CD4^+^ and CD8^+^ T cell (**a, d**), indicated intracellular proteins (**b, e**), and surface markers (**c, f**) were determined by flow cytometer. Data are shown as mean ± SEM, *n* = 3 biologically independent samples, significance was calculated by unpaired Two-tail Student’s t-test. *ns*, no significant differences.

**Extended Data Fig. 9:**
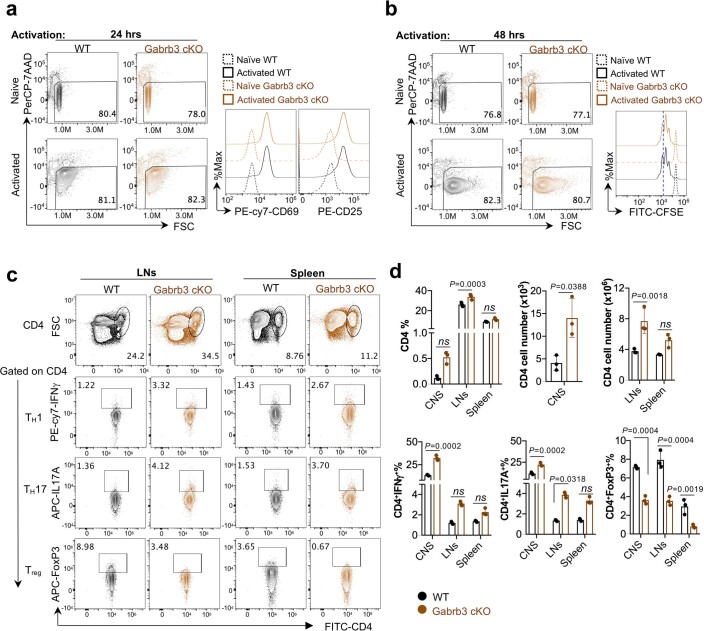
GABA receptor is required for T cell differentiation but not activation. **a, b**, Cell viability, cell activation markers (**a**), and CFSE dilution (**b**) were determined by flow cytometry (*n* = 3 biologically independent experiments). **c**, T cells were isolated from indicated sites in experimental animals described **in** Fig. 5g. The expression of indicated proteins was determined by flow cytometry. **d**, Statistical analysis (*n* = 3 biologically independent samples) was calculated by unpaired Two-tail Student’s t-test. Data are shown as mean ± SEM, *ns*, no significant differences. EAE: experimental autoimmune encephalomyelitis, CNS: central nervous system, LNs: lymph nodes.

In line with earlier studies^14^, we have found that T_eff_ cells express various subunits of the GABA_A_ receptor (GABA_A_-R) (Fig. 1f). Additionally, T cells can produce and secrete a large amount of GAB into the extracellular compartment, which may elicit a context-dependent autocrine signalling response to regulate T cell differentiation (Fig. 5a). Supporting this idea, a low level of GAB supplementation could reduce T_H_17 but enhance iT_reg_ differentiation without significantly affecting T cell activation and proliferation in vitro (Fig. 5b,c and Extended Data Fig. 7a,b). Conversely, GABA_A_-R antagonists with distinct antagonistic mechanisms enhanced T_H_17 cell differentiation but reduced iT_reg_ cell differentiation without affecting T cell activation and proliferation in vitro (Fig. 5c–e and Extended Data Fig. 7c,d). The β-subunit is a core component of GABA_A_-R, and the β3 subunit (encoded by *Gabrb3*) was highly expressed in all T_eff_ subsets (Fig. 1f). We generated a T cell-specific *Gabrb3-*knockout strain (*Gabrb3* cKO) by crossing the *Gabrb3*^*fl*^ strain with the *CD4*-Cre strain. *Gabrb3* deletion did not result in T cell development defects in the thymus, spleen and lymph nodes (Extended Data Fig. 8a–f). In addition, cell viability, the expression of cell surface activation markers and cell proliferation were comparable in both WT and *Gabrb3* cKO T cells after activation in vitro (Extended Data Fig. 9a,b). However, genetic ablation of *Gabrb3* promoted pro-inflammatory T_H_17 cell differentiation while reducing anti-inflammatory iT_reg_ cell differentiation in vitro (Fig. 5c,f). Notably, GABA supplementation only affected WT but not *Gabrb3* cKO T cell differentiation in vitro (Fig. 5c,f). Finally, the T cell-specific *Gabrb3* deletion let to significantly deteriorated EAE pathogenic progression, associated with increased inflammatory CD4^+^ T cells and decreased FoxP3^+^CD4^+^ T cells in the CNS and periphery (Fig. 5g,h and Extended Data Fig. 9c,d).

### GAB regulates T cells through a bimodal mechanism of action

**Fig. 6:**
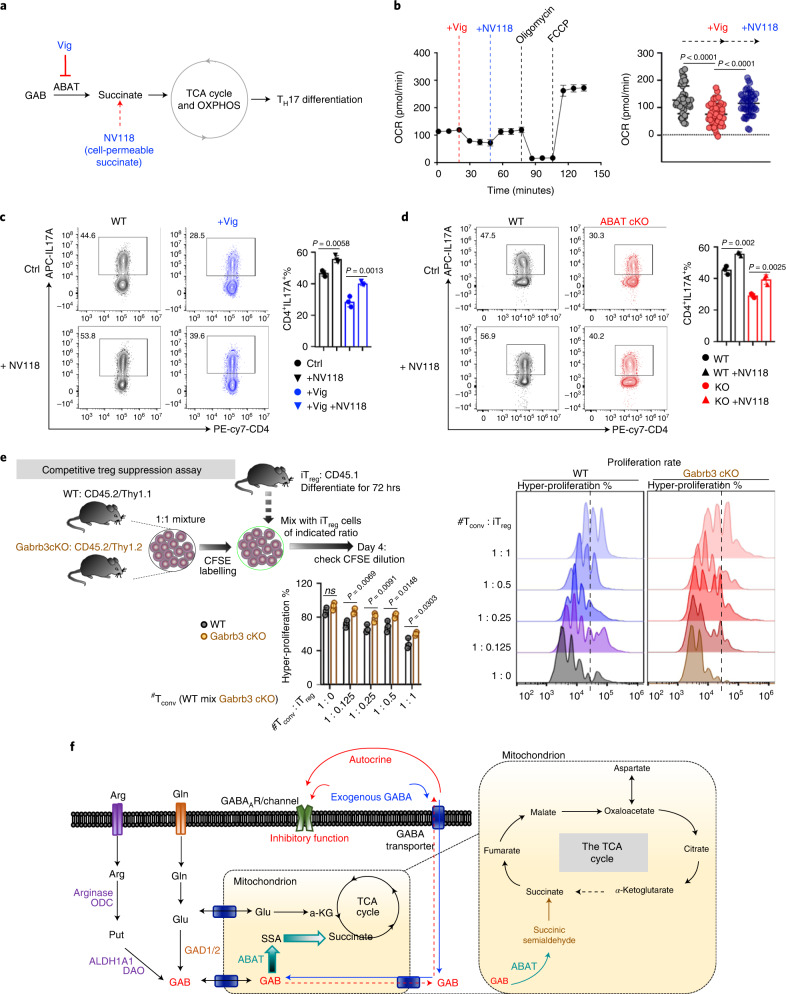
GAB exerts both bioenergetic and receptor signalling-mediated control of T cell differentiation. **a**, Schematic diagram of GAB catabolism through the TCA cycle. **b**, OCR of T_H_17 cells with the indicated treatments determined by Seahorse (*n* = 16 independent biological samples from *n* = 3 independent biological experiments); significance was calculated by two-way ANOVA with Sidak’s multiple-comparisons test. **c**,**d**, Expression of IL-17A in the indicated groups determined by flow cytometry (*n* = 3 independent biological samples); significance was determined by one-way ANOVA with Tukey’s multiple-comparisons test. **e**, Left, experimental diagram of the competitive T_reg_ suppression assay. Right, CFSE dilution was determined by flow cytometry (*n* = 3 independent biological samples); significance was determined by two-way ANOVA with Sidak’s multiple-comparisons test. **f**, Schematic conceptual model in which GAB generated by Gln and Arg can regulate T cell differentiation by entering the TCA cycle or being exporting into the extracellular environment and acting on GABA_A_-R. T_conv_, conventional T cells.

**Extended Data Fig. 10:**
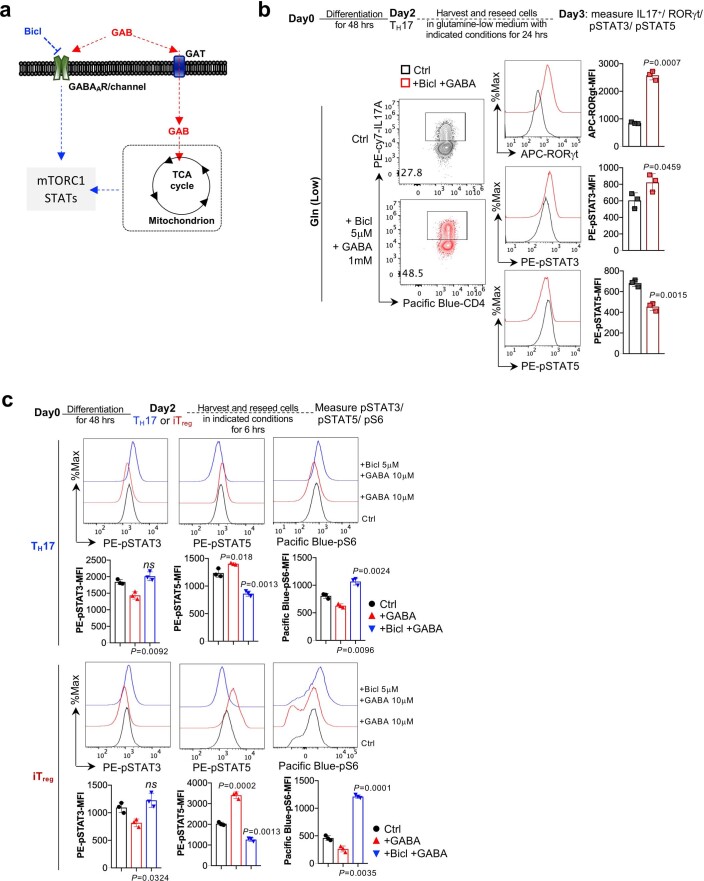
GAB controls T cell signaling pathways through both receptor and mitochondrial metabolism. **a**, Schematic diagram of GAB metabolism and GABAA-R-mediated signaling response. **b, c**, The schematic diagram of the experiment (top), the expression of indicated proteins from each group was determined by flow cytometry (*n* = 3 biologically independent samples). Data are shown as mean ± SEM. **b**, Significance was calculated by unpaired Two-tail Student’s t-test. **c**, Significance was calculated by one-way ANOVA with Tukey’s multiple comparisons test. *ns*, no significant differences.

Next, we sought to dissect the effect of ABAT-dependent mitochondrial anaplerosis and GABA_A_-R-mediated signalling on T cell differentiation and function (Extended Data Fig. 10a). We envisioned that the ABAT-dependent anaplerotic reaction might support T_H_17 differentiation by providing succinate to fuel mitochondrial OXPHOS (Fig. 6a). Indeed, inhibiting ABAT activity by Vig suppressed oxygen consumption, which was reversed by adding a cell-permeable succinate analogue NV118 (Fig. 6a,b)^24^. In line with the effect of NV118 on oxygen consumption, the NV118 supplementation could partially reverse the inhibition of T_H_17 differentiation resulting from genetic or pharmacological inhibition of ABAT (Fig. 6c,d). Next, we asked whether ABAT-dependent mitochondrial anaplerosis could impact transcription factors critical for T_H_17 lineage differentiation, such as RORγt and STATs^25^. To test this idea, we reduced the medium’s Gln concentration and added a high concentration of GAB (1 mM) with a GABA_A_-R antagonist. We reasoned that reducing Gln levels would force cells to use GAB as a mitochondrial fuel and adding the receptor antagonist would eliminate the effects of receptor signaling. Indeed, GAB supplementation significantly enhanced the levels of RORγt and phosphorylated STAT3 (pSTAT3) but reduced the levels of phosphorylated STAT5 (pSTAT5) (Extended Data Fig. 10b).

Next, we sought to determine whether modulating GABA_A_-R affects key signalling molecules involved in regulating T_H_17 and iT_reg_ differentiation. We treated T cells with a low dose of GAB (10 μM) in the presence of a GABA_A_-R antagonist. We reasoned that the low dose of GAB could engage the receptor-mediated signalling response without significantly fuelling mitochondrial metabolism. We assessed the levels of phosphorylated STAT proteins and the phosphorylation of a canonical mTORC1 substrate (pS6) because mTORC1 is critical for determining T_H_17 and iT_reg_ differentiation^26,27^. Treating T_H_17 and iT_reg_ cells with a low dose of GAB suppressed pSTAT3 and mTORC1 substrate phosphorylation (pS6) but increased pSTAT5 (Extended Data Fig. 10c). Notably, the effects of GAB on these signalling molecules could be reversed by a GABA_A_-R antagonist (Extended Data Fig. 10c). We showed that iT_reg_ cells can excrete GAB into the extracellular compartment (Extended Data Fig. 1g–i). Finally, we sought to determine whether GAB contributes to T_reg_-dependent immune suppression. We performed a competitive T_reg_ suppression assay by co-culturing iT_reg_ cells with WT and *Gabrb3* cKO CD4^+^ T cells that carried different isogenic markers (Fig. 6e). Indeed, *Gabrb3* cKO CD4^+^ T cells proliferated better than the WT group, indicating that genetic ablation of GABA_A_-R could partially alleviate iT_reg_-mediated suppression (Fig. 6e). Together, these results suggest that GAB is an abundant metabolite produced by T cells and exerts both bioenergetic control and receptor-mediated signalling control of T cell differentiation (Fig. 6f).

## Discussion

The vertebrate immune and nervous systems are intimately connected with each other developmentally, anatomically and physiologically. Interaction between the two systems coordinates their sensory functions to ensure organismal homeostasis and survival^28–31^. Immune cells and neurons can communicate with each other through a group of shared ligand molecules and receptors, including the neurotransmitter GABA and its receptors^14,32^. Beyond mediating intersystem communication between the immune and nervous systems, growing evidence suggests that GABA can also act as a paracrine signalling molecule mediating intrasystem communication to regulate immune response^33^. One recent study has found that B cells can produce GABA and suppress anti-tumour immunity through paracrine modulation of intratumoural macrophages and CD8^+^ T cells^34^. Additionally, GABA in macrophages has been implicated as an intracellular metabolite with a pro-inflammatory function^14^. Here, we show that GAB (the biochemical form of GABA at physiological pH) is one of the most abundant metabolites in T cells and promotes inflammation through modulating T cell proliferation and differentiation. Depending on the status of its catabolizing enzyme ABAT, GAB can act as a conditional anaplerotic substrate to promote T_H_17 cell differentiation or an autocrine signalling metabolite to enhance iT_reg_ cell differentiation. In addition to its role in mediating intercellular communications, GAB also serves as a metabolic and signalling gatekeeper to regulate inflammation in a T cell-autonomous manner.

T_eff_ cells consume Gln and Arg at high rates^35,36^. Beyond a general requirement for protein synthesis, Gln and Arg support T cell proliferation and function through their catabolic products. Gln is a primary carbon source to sustain the TCA cycle, which generates energy through OXPHOS and allocates carbon to produce biosynthetic precursors to support T cell growth^21,36,37^. Similarly, Arg catabolism is coupled with the urea cycle to produce bioactive metabolites such as polyamines to support T cell proliferation and differentiation^10–12^. Our results show that both Arg catabolism (via Put) and Gln catabolism (via Glu) are coupled with GAB biosynthesis in T_H_17 cells, implicating GAB as a crucial metabolic node and a branch point in amino acid catabolism. GAB can be consumed through the TCA cycle to enhance bioenergetic and biosynthetic capacities or be secreted as an autocrine signalling metabolite depending on the status of ABAT. We have further revealed that Gln can replenish the TCA cycle intermediate metabolites through either Glu or GAB anaplerosis. Glu increases the levels of α-ketoglutarate (α-KG), while GAB increases the levels of succinate. Therefore, it is conceivable that the carbon input from Glu or GAB may change the intracellular α-KG to succinate ratio reciprocally. Hence, the GABA shunt in T cells may impact the hypoxia signalling response and/or DNA/histone methylation patterns by modulating the enzymatic activities of the α-KG-dependent dioxygenase family^37–40^, and Glu and Put are highly abundant intracellular metabolites that can be secreted to the extracellular environment by T_H_17 cells (Fig. 1a)^10,41^. The GAB-catabolizing enzyme ABAT may provide a sensitive and precise regulation of the three interconnected and highly abundant metabolites: GAB, Glu and Put, permitting rapid metabolic and signalling responses to control inflammation.

The high and dynamic metabolic demands of T cells during inflammatory and autoimmune responses require fine-tuned regulation of central carbon and ancillary metabolic pathways. Hence, metabolic pathways have been therapeutically exploited to target inflammatory and autoimmune diseases^42,43^. Disruption of central carbon catabolism can affect many cellular processes and cell types. However, targeting ancillary metabolic pathways engaged in a small group of specialized immune cells under physio-pathological conditions may result in less toxicity but maximal clinical benefits^6^. Gene and protein expression profiling studies have suggested that human autoimmune diseases, including MS, type 1 diabetes and rheumatoid arthritis, are associated with the dysregulation of GABA-related metabolic and signaling genes^44–47^. Interestingly, cortical GAB levels are lower in patients with relapsing–remitting multiple sclerosis MS than in healthy controls^48,49^. In addition, one recent study based on genome-scale metabolic modelling and in silico simulations for drug response indicated that GAB metabolism and signalling pathway not only are involved in the disease process but also are potential drug targets in human autoimmune diseases^50^. Consistent with clinical profiling and in silico studies, pharmacological modulation of GAB metabolism and receptor-mediated signalling response could ameliorate pathological phenotypes in several preclinical models of autoimmune diseases^51–55^. Our results further elucidate a previously unrecognized aspect of the T cell-intrinsic effects conferred by GAB catabolism and receptor-mediated signalling. Collectively, GAB-modulating strategies via blockade of GAB catabolism, activation of receptor-mediated response, or both may present a promising therapy for treating inflammatory and autoimmune diseases.

## Methods

### Mice

C57BL/6 (WT), Flippase (B6.129S4*Gt(ROSA)26Sor*^*tm1(FLP1)Dym*^/RainJ), OT-II (B6.Cg-Tg(TcraTcrb)425Cbn/J), CD45.1^+^ (B6.SJL-*Ptprc*^*a*^*Pepc*^*b*^/BoyJ), *Rag1*^−/−^ (B6.129S7-*Rag1*^*tm1Mom*^/J), IL17A-IRES-GFP-KI (C57BL/6-*Il17a*^*tm1Bcgen*^/J), FoxP3^GFP+^ (C57BL/6-Tg(Foxp3-GFP)90Pkraj/J) and *Gabrb3*^*fl*^ (B6;129-*Gabrb3*^*tm2.1Geh*^/J) mice were obtained from the Jackson Laboratory (JAX, Bar Harbor, ME). Mice with one targeted allele of *Abat* on the C57BL/6 background (*Abat*^tm1a(EUCOMM)Hmgu^) were generated by the European Conditional Mouse Mutagenesis Program (EUCOMM)^56^. The mice were first crossed with a transgenic Flippase strain (B6.129S4*Gt(ROSA)26Sor*^*tm1(FLP1)Dym*^/RainJ) to remove the *lacZ*-reporter allele and then crossed with the *Cd4*-Cre strain to generate the T cell-specific *Abat* knockout strain (*Abat* cKO). OT-II mice were crossed with *Cd4*-Cre *Abat* cKO mice to generate the OT-II *Cd4*-Cre *Abat* cKO mice. OT-II mice were crossed with *Thy1.1*^+^ mice (B6.PL-*Thy1*^a^/CyJ) to generate the OT-II *Thy1.1* mice. *Gabrb3*^*fl*^ mice were crossed with the *Cd4*-Cre strain to generate T cell-specific *Gabrb3-*knockout strain (*Gabrb3* cKO). For one independent experiment, we used male and female mice from the same strain that were both sex and age matched (6–12 weeks old), such as two males and two females for WT mice, as well as for KO mice. All mice were bred and kept in specific pathogen-free conditions at the Animal Center of the Abigail Wexner Research Institute at Nationwide Children’s Hospital. A low-fat diet was provided (Envigo 2920, the irradiated form of 2020X; https://insights.envigo.com/hubfs/resources/data-sheets/2020x-datasheet-0915.pdf). Animals were killed by carbon dioxide asphyxiation followed by cervical dislocation under protocols approved by the Institutional Animal Care and Use Committee of the Abigail Wexner Research Institute at Nationwide Children’s Hospital (IACUC; protocol number AR13-00055).

### Murine T cell isolation and culture

Naive CD4^+^ T cells were enriched from mouse spleen and lymph nodes by negative selection using the MojoSort™ Mouse CD4^+^ Naive T Cell Isolation Kit (MojoSort, BioLegend) according to the manufacturer’s instructions. For the activation assay, freshly isolated CD4^+^ T cells were either maintained in a culture medium with 5 ng/ml^-1^ IL-7 or activated with 5 ng/ml^-1^ IL-2 and plate-bound anti-mouse CD3 and anti-mouse CD28. The culture plates were precoated with 2 μg/ml^-1^ anti-mouse CD3 and 2 μg/ml^-1^ anti-mouse CD28 antibodies overnight at 4 °C. Naive tT_reg_ cells were enriched from mouse spleen and lymph nodes by positive selection using the MojoSort™ Mouse CD4^+^CD25^+^ Regulatory T Cell Isolation Kit (MojoSort, BioLegend) according to the manufacturer’s instructions. For the activation assay, freshly isolated CD4^+^CD25^+^ regulatory T cells were either maintained in a culture medium with 5 ng/ml^-1^ IL-2 or activated with 5 ng/ml^-1^ IL-2 and anti-mouse CD3/CD28 beads according to the manufacturer’s instructions (Gibco, Thermo Fisher Scientific). Unless indicated separately, the cells were seeded in the RPMI-1640 medium (Corning) supplemented with 10% FBS, or heat-inactivated dialysed FBS (DFBS), 2 mM l-glutamine, 1% sodium pyruvate (Sigma-Aldrich), 100 units/ml^-1^ penicillin, 100 μg/ml^-1^ streptomycin and 0.05 mM 2-mercaptoethanol (Sigma-Aldrich) at 37 °C and 5% CO_2_.

For CD4^+^ T cell differentiation, 48-well culture plates were precoated with 2 μg/ml^-1^ (iT_reg_ differentiation), 5 μg/ml^-1^ (T_H_1/ T_H_2 differentiation) or 10 μg/ml^-1^ (T_H_17 differentiation) anti-mouse CD3 and anti-mouse CD28 antibodies overnight at 4 °C. Freshly isolated naive CD4^+^ T cells (0.5 × 10^6^ cells per ml) were activated with plate-bound antibodies and with mouse IL-2 (3 ng/ml^-1^) and human TGF-β1 (10 ng/ml^-1^) for iT_reg_ differentiation, with mouse IL-2 (10 ng/ml^-1^) and mouse IL-12 (20 ng/ml^-1^) for T_H_1 differentiation, with mouse IL-2 (2 ng/ml^-1^), mouse IL-4 (50 ng/ml^-1^) and anti-mouse IFN-γ (10 μg/ml^-1^) for T_H_2 differentiation, or with mouse IL-6 (50 ng/ml^-1^), human TGF-β1 (20 ng/ml^-1^), anti-mouse IL-2 (8 μg/ml^-1^), anti-mouse IL-4 (8 μg/ml^-1^), and anti-mouse IFN-γ (8 μg/ml^-1^) for T_H_17 differentiation. In some experiments, Vig (1 mM), GABA (0.1 μM~1 mM), NV118 (25 μM), GABA_A_-R antagonists including bicuculline (Bicl, 5 or 50 μM), picrotoxin (PicroT, 5 or 50 μM) and flumazenil (10 or 1 μM), R162 (20 μM), oligomycin (1.5 μM), FCCP (1 μM), or AG (0.2 mM) was added to cell culture medium. Additional information on cytokines, antibodies and chemicals is listed in Supplementary Table 1.

### Flow cytometry

For analysing surface markers, cells were stained in phosphate-buffered saline (PBS) containing 2% (wt/vol) BSA and the appropriate antibodies from BioLegend. For analysing the intracellular cytokines IFN-γ and IL-17A, T cells were stimulated for 4 hrs with eBioscience™ Cell Stimulation Cocktail (eBioscience) before being stained with cell surface antibodies. Cells were then fixed and permeabilized using FoxP3 Fixation/Permeabilization solution according to the manufacturer’s instructions (eBioscience). Cell proliferation was assessed using CFSE staining according to the manufacturer’s instructions (Invitrogen). Cell viability was evaluated by 7AAD staining according to the manufacturer’s instructions (BioLegend). For analysing DNA/RNA content, cells were collected and stained for surface markers before being fixed with 4% paraformaldehyde for 30 min at 4 °C, followed by a permeabilization step with FoxP3 permeabilization solution (eBioscience). Cells were stained with 7AAD for 5 min and then stained with pyronin-Y (4 μg/ml^-1^; PE) for 30 min before being analysed using flow cytometer with the PerCP channel for 7AAD (DNA) and PE channel for pyronin-Y (RNA). A protein synthesis assay kit (Item No.601100, Cayman) was used for analysing protein content. Briefly, cells were incubated with *O*-propargyl-puromycin (OPP) for 1 hr and then they were fixed and stained with 5 FAM-azide staining solutions before being analysed using a flow cytometer with the FITC channel. For analysing the cell cycle profile, cells were incubated with 10 μg/ml^-1^ BrdU for 1 hr, followed by cell surface staining, fixation and permeabilization based on the Phase-Flow Alexa Fluor 647 BrdU Kit (BioLegend). Flow cytometry data were acquired on Novocyte (ACEA Biosciences) and were analysed with FlowJo software (TreeStar). Additional information on flow cytometry antibodies is listed in Supplementary Table 2.

### T_reg_ cell suppression assay

For the iT_reg_ suppression assay, naive CD4^+^ T cells isolated from CD45.1 mice using the naive CD4^+^ mouse T cell isolation kit (BioLegend) were differentiated for 3 d to generate iT_reg_ cells. Naive CD4^+^ T cells isolated from CD45.2/*Thy1.1* WT donor mice and CD45.2/*Thy1.2 Gabrb3* cKO donor mice were mixed at a 1:1 ratio (as T_conv_ cells) and labelled with CFSE. Then, approximately 5 × 10^4^ T_conv_ cells were mixed with iT_reg_ cells (with indicated ratios) and cultured with 3 ng/ml^-1^ IL-2 and anti-mouse CD3/CD28 beads. Cells were collected 4 d later and processed to assess proliferation by flow cytometry analysis.

### Retrovirus production and transduction

Phoenix Eco cells that were cultured in fresh DMEM media (Corning) supplemented with 10% heat-inactivated FBS and 0.5% penicillin-streptomycin were transfected with the control plasmid (pMIC, MSCV-IRES-mCherry) or pMIC-ABAT (Supplementary Table 3). Viral-Boost Reagent (ALSTEM) was added to the culture medium at a 1:600 dilution 6 h after transfection. Cell medium was collected at 48 h after transfection, centrifuged at 300*g* for 10 min, and then filtered through a 0.45-μm filter unit (GVS Filter Technology). Retrovirus Precipitation Solution (ALSTEM) was added to retrovirus-containing supernatant at 1:4 dilution and incubated overnight at 4 °C, followed by centrifugation at 1,500*g* for 30 min at 4 °C to concentrate the virus. Then, approximately 0.3 × 10^6^ activated CD4^+^ T cells (1 d after activation) were resuspended in 1 ml of retroviral supernatant containing 8 µl/ml^-1^ Lipofectamine (Invitrogen) and cultured under iT_reg_ differentiation for 4 d.

### Adoptive cell transfer assays

For homeostatic proliferation in lymphopenic *Rag*^−/−^ mice, naive CD4^+^ T cells isolated from donor mice using a naive CD4^+^ mouse T cell isolation kit (BioLegend) were labelled with CFSE. Approximately 1 × 10^7^ cells (mix of WT and KO cells at a 1:1 ratio) in 150 μl of PBS were transferred via caudal venous injection into 6- to 8-week-old sex-matched host mice. Mice were killed between 4–7 d after cell transfer. Lymph nodes and spleen were collected and processed to assess cell ratio and proliferation by flow cytometry analysis.

For antigen-driven proliferation using OT-II mice, naive CD4^+^ T cells isolated from OT-II/CD45.2 TCR-transgenic donor mice using the naive CD4^+^ mouse T cell isolation kit (BioLegend) were labelled with CFSE. Approximately 1 × 10^7^ cells (mix of WT and KO cells at a 1:1 ratio) in 150 μl of PBS were transferred via caudal venous injection into 6- to 8-week-old sex-matched CD45.1 host mice. Host mice were immunized subcutaneously in the hock area (50 μl each site) in both legs with 1 mg/ml^-1^ ovalbumin (OVA)^323–339^ peptide (InvivoGen) emulsified with complete Freund adjuvant (CFA; InvivoGen). The mice were then killed 8 d after immunization. Lymph nodes were collected and processed to assess cell ratio, proliferation and protein expression by flow cytometry analysis.

### EAE

Mice were immunized subcutaneously with 100 μg of myelin oligodendrocyte glycoprotein (MOG)_35–55_ peptide emulsified in CFA, which was made from IFA (Difco) plus *Mycobacterium tuberculosis* (Difco). Mice were injected intraperitoneally with 200 ng of pertussis toxin (PTX, List Biological Laboratories) on the day of immunization and 2 d later. In the experiments shown in Fig. 4j,k and Extended Data Fig. 5c,d, the mice were injected intraperitoneally with 250 mg/kg^-1^ of Vig in 100 μl PBS daily from day 3 after immunization throughout the end of the experiment. In the experiments shown in Fig. 5g,h and Extended Data Fig. 9c,d, the animals were injected with PTX only once on the day of immunization for a suboptimal EAE induction. All mice were observed daily for clinical signs and scored as described previously^10^. In some experiments, the mice were killed when the control mice reached the onset of symptoms. The CNS (brain and spinal cord), spleen and peripheral lymph nodes were collected and mashed to generate the single-cell suspension. The cell suspension was centrifuged on a 30%/70% Percoll gradient at 500*g* for 30 min to isolate mononuclear cells from the CNS, followed by cell surface and intracellular staining and flow cytometry analysis described above.

### Stable isotope labelling experiments

#### [^13^C_5_]Gln, [^13^C_6_]Arg and [^13^C_6_]Glc labelling of T_H_17 cells

Naive CD4^+^ T cells isolated from WT mice were polarized for 72 h under T_H_17 culture conditions before being collected and reseeded at 2 × 10^6^ cells per ml in a conditional medium (RPMI-1640) containing 4 mM [^13^C_5_]Gln, 1 mM [^13^C_6_]Arg or 10 mM [^13^C_6_]Glc. After 12 h of culture, around 1 × 10^7^ cells for each sample were collected and washed three times with PBS before being snap-frozen.

#### [^13^C_6_]Arg labelling of T_H_17 cells

T_H_17 cells (as described above) were pretreated with vehicle or Vig (1 mM) for 1 h before being collected and reseeded at a density of 2 × 10^6^ cells per ml in a conditional medium (RPMI-1640) containing 4 mM 1 mM [^13^C_6_]Arg with vehicle or Vig (1 mM). After 6 h of culture, around 1 × 10^7^ cells for each sample were collected and washed three times with PBS before being snap-frozen.

#### [^13^C_4_]Put labelling of T_H_17 cells

T_H_17 cells (as described above) were pretreated with vehicle, Vig (1 mM), or AG (0.2 mM) for 1 h and then collected and reseeded at a density of 2 × 10^6^ cells per ml in a conditional medium (RPMI-1640) containing 0.1 mM [^13^C_4_]Put and 10 µM Arg and with vehicle, Vig (1 mM), or AG (0.2 mM) treatment. After 6 h of culture, around 1 × 10^7^ cells for each sample were collected and washed three times with PBS before being snap-frozen.

#### [^13^C_4_]GABA labelling of T_H_17, iT_reg_ and T_H_1 cells

Naive CD4^+^ T cells isolated from WT mice were polarized for 72 h under T_H_17, iT_reg_ or T_H_1 culture conditions before being collected and re-seeded at a density of 2 × 10^6^ cells per ml in a conditional medium (RPMI-1640) containing 0.5 mM [^13^C_4_]GABA, 0.1 mM Gln and the GABA_A_-R antagonist bicuculline (5 µM). After 12 h of culture, around 1 × 10^7^ cells for each sample were collected and washed three times with PBS before being snap-frozen.

#### [^13^C_4_]GABA labelling of T_H_17 cells with Vig

T_H_17 cells (as described above) generated from WT or *Abat* cKO mice were pretreated with vehicle or Vig (1 mM) for 1 h before being collected and reseeded at a density of 2 × 10^6^ cells per ml in the conditional medium containing 0.5 mM [^13^C_4_]GABA, 0.1 mM Gln and the GABA_A_-R antagonist bicuculline (5 µM) and with vehicle or Vig (1 mM) treatment. After 12 h of culture, around 1 × 10^7^ cells for each sample were collected and washed three times with PBS before being snap-frozen.

#### [^13^C_5_]Gln labelling of T_H_17 cells with multiple inhibitors

T_H_17 cells (as described above) were pretreated with vehicle, Vig (1 mM) or R162 (20 μM) for 1 h and then collected and reseeded at a density of 2 × 10^6^ cells per ml in a conditional medium (RPMI-1640) containing 4 mM [^13^C_5_]Gln and with vehicle, Vig (1 mM), R162 (20 μM) or the combination of Vig and R162 treatment. After 6 h of culture, around 1 × 10^7^ cells for each sample were collected and washed three times with PBS before being snap-frozen. Additional information on stable isotope labelling is listed in Supplementary Table 4.

### Gas chromatography–mass spectrometry sample preparation and analysis

GC–MS was performed as previously described^57^, and cell pellets were resuspended in 0.45 ml of −20 °C methanol/water (1:1 v/v) containing 20 µM l-norvaline as internal standard. Further extraction was performed by adding 0.225 ml of chloroform followed by vortexing and centrifugation at 15,000*g* for 5 min at 4 °C. The upper aqueous phase was evaporated under vacuum using a Speedvac centrifugal evaporator. Separate tubes containing varying amounts of standards were evaporated. Dried samples and standards were dissolved in 30 μl of 20 mg/ml^-1^ isobutylhydroxylamine hydrochloride (TCI #I0387) in pyridine and incubated for 20 min at 80 °C. An equal volume of *N*-tert-butyldimethylsilyl-*N*-methyltrifluoroacetamide (MTBSTFA) (Soltec Ventures) was added and incubated for 60 min at 80 °C. After derivatization, samples and standards were analysed by GC–MS using an Rxi-5ms column (15 m × 0.25 internal diameter × 0.25 μm, Restek) installed in a Shimadzu QP-2010 Plus GC–MS instrument. GC–MS was programmed with an injection temperature of 250 °C, injection volume of 1.0 µl and a split ratio of 1/10. The GC oven temperature was initially 130 °C for 4 min, rising to 250 °C at 6 °C min^-1^ and to 280 °C at 60 °C min^-1^ with a final hold at this temperature for 2 min. GC flow rate, with helium as the carrier gas, was 50 cm s^-1^. GC–MS interface temperature was 300 °C, and the (electron impact) ion source temperature was 200 °C, with an ionization voltage of 70 eV. Fractional labelling from ^13^C-labelled substrates and mass isotopomer distributions were calculated as described previously^57^. Data from standards were used to construct standard curves in MetaQuant^58^, from which metabolite amounts in samples were calculated. Metabolite amounts were corrected for the recovery of the internal standard and for ^13^C labelling to yield total (labelled and unlabelled) quantities in nanomoles per sample and then adjusted by cell number.

### Liquid chromatography–mass spectrometry sample preparation and analysis

Naive CD4^+^ T cells were polarized under T_H_0, T_H_1, T_H_17 and iT_reg_ culture conditions or cultured with IL-7 (T_nai_ condition) for 72 h. Then, the cells were collected, washed with PBS and reseeded at a density of 5 × 10^6^ cells per ml in fresh medium. After 6 h of culture, the cell medium was collected and snap-frozen. Sample preparation and analysis were carried out as described previously at Metabolon^59^. In brief, sample preparation involved protein precipitation and removal with methanol, shaking and centrifugation. The resulting extracts were profiled on an accurate mass global metabolomics platform consisting of multiple arms differing by chromatography methods and MS ionization modes to achieve broad coverage of compounds differing by physiochemical properties such as mass, charge, chromatography separation and ionization behaviour. Metabolites were identified by automated comparison of the ion features in the experimental samples to a reference library of chemical standard entries that included retention time, molecular weight (*m/z*), preferred adducts and in-source fragments as well as associated MS spectra and were curated by visual inspection for quality control using a software developed at Metabolon.

### Metabolite quantification

In some experiments, T_H_17 cells were suspended at a density of 5 × 10^6^ cells per ml with medium containing vehicle or Vig (1 mM). After 6 h of culture, blank medium (without cells) and spent medium were collected. The levels of Gln and Glu were measured using the Bioanalyzer (YSI 2900). Following the manufacturer’s instructions, Arg and GAB quantities were determined by l-Arginine Assay Kit (BioVision) and GABA Research ELISA Kit (LDN). Consumption or production of each metabolite was determined by calculating the difference between blank and spent media.

### OCR

Following the manufacturer’s instructions, the OCR was determined using the Seahorse XFe96 Analyzer (Agilent Technologies). Briefly, approximately 1 × 10^5^ T_H_17 cells were suspended in a 50 µl assay medium (Seahorse XF RPMI Assay Medium, pH 7.4, Agilent Technologies) containing 10 mM Glc, 2 mM Glu and 1 mM pyruvate and were seeded in an XF96 Cell Culture Microplates (Seahorse, Agilent Technologies) precoated with poly(d-lysine) (50 µg ml^-1^; Millipore). The cells were centrifuged at 200*g* for 2 min on a zero-braking setting to immobilize the cells before they were supplied with an additional 130 µl of assay medium and kept in a non-CO_2_ incubator for 30 min. Data analysis was performed using the Seahorse Wave Software (Seahorse, Agilent Technologies). In some experiments, the GABA_A_-R antagonist bicuculline (5 µM) was added along with GABA to prevent the activation of GABA_A_-R. Various compounds were injected into each well sequentially to achieve the following final concentrations: 0.5 mM GABA, 1 mM Vig, 20 µM R162, 1.5 µM oligomycin, and 1 µM FCCP.

### Western blot analysis, RNA extraction, qPCR, and RNA-seq and NMR analysis of medium

Details are provided in the Supplementary Information.

### Statistical analysis

Statistical analysis was conducted using the GraphPad Prism software (GraphPad Software; v 8.0.1). To determine the statistical significance, different tests including unpaired two-tailed Student’s *t*-test, one-way ANOVA with Tukey’s multiple-comparisons test and two-way ANOVA with Sidak’s multiple-comparisons test were used as indicated in the figure legends. The number of experimental repeats is indicated in the figure legends. R software (v 4.2.1) was used for Metabolon and RNA-seq data analysis. *P* values that were considered significant are shown in the corresponding figures.

### Reporting summary

Further information on the research design is available in the Nature Research Reporting Summary linked to this article.

## Supplementary information

Supplementary Information Supplementary Methods, Supplementary Tables 1–5 and Supplementary Figures 1–2.

Reporting Summary

## Data Availability

The RNA-seq datasets generated for this study can be found in the Gene Expression Omnibus under accession GSE190818. The authors declare that all other data supporting the findings of this study are available within the paper and supplementary information files. Source data are provided with this paper.

## Source data

Source Data Fig. 1 Metabolomic data for amino acid meta of Fig. 1b. GC–MS-based heatmap data for cell medium of Fig. 1c. GC–MS-based heatmap data for cell pellet of Fig. 1c. qPCR data of Fig. 1f. GC–MS-based heatmap data for cell medium of Fig. 1k. GC–MS-based heatmap data for cell pellet of Fig. 1k
Source Data Fig. 2 Unprocessed Western blots for Fig. 2a.
Source Data Fig. 2 qPCR data for Fig. 2e.
Source Data Fig. 4 Unprocessed Western blots for Fig. 4a.
Source Data Fig. 4 RNA-seq data for the heatmap for Fig. 4d. RNA-seq data IPA for Fig. 4f. RNA-seq data (all data) for Fig. 4d–f.
Source Data Extended Data Fig. 1 Metabolomic data (all metabolites) for Extended Data Fig. 1b–d. Metabolomic data (energy-meta) for Extended Data Fig. 1e. Metabolomic data (nucleotide-meta) for Extended Data Fig. 1f. Metabolomic data (lipid-meta) for Extended Data Fig. 1g.
