# Supplementary material for: GAB functions as a bioenergetic and signalling gatekeeper to control T cell inflammation
Source: Nat Metab. 2022 Oct 3;4(10):1322–35. doi: 10.1038/s42255-022-00638-1 (PMC9584824; doi:10.1038/s42255-022-00638-1)
Supplement: Supplementary file 1 — Supplementary Methods, Supplementary Tables 1–5 and Supplementary Figures 1–2. [file 42255_2022_638_MOESM1_ESM.pdf]

nature metabolism

Article https://doi.org/10.1038/s42255-022-00638-1

GAB functions as a bioenergetic and signalling gatekeeper to control T cell inflammation

In the format provided by the authors and unedited

Supplementary information

Materials and Methods

Western Blot Analysis

For protein extraction, cells were lysed and sonicated at 4°C in a lysis buffer (50 mM Tris-HCl, pH 7.4, 150 mM NaCl, 0.5% SDS, 5 mM sodium pyrophosphate, protease, and phosphatase inhibitor tablet), then centrifuged at 13,000 ×*g* for 15 min for recovering. The samples were boiled in the mixture of LDS Sample Buffer (NuPAGE) and Reducing solution (Thermo Fisher Scientific) for 5 min, after transferring to PVDF membranes by using the iBlot Gel Transfer Device (Thermo Fisher Scientific), then incubated with primary anti-ABAT (clone B-12, Santa Cruz Biotechnology) followed by incubating with the secondary antibodies conjugated with horseradish peroxidase. Immunoblots were developed on films using the enhanced chemiluminescence technique.

RNA Extraction, qPCR, and RNAseq

Total RNA was isolated by using the RNeasy Mini Kit (Qiagen). The cDNA synthesis was processed using Random hexamers and M-MLV Reverse Transcriptase (Invitrogen). BIO-RAD CFX284^{TM} Real-Time PCR Detection System was used for SYBR green-based quantitative PCR. The relative gene expression was determined by the comparative *CT* method, also referred to as the $2^{-\Delta\Delta CT}$ method. The data were presented as the fold change in gene expression normalized to an internal reference gene (beta2-microglobulin) and relative to the control (the first sample in the group). Fold change = $2^{-\Delta\Delta C}{}_{\mathrm{T}}$ = [($CT_{\text{gene of interest}}$- $CT_{\text{internal reference}}$)] sample A - [($CT_{\text{gene of interest}}$- $CT_{\text{internal reference}}$)] sample B. Samples for each experimental condition were run in triplicated PCR reactions. Primer sequences were obtained from Primer Bank to detect target genes (**Supplementary Table 5**).

For RNA sequencing analysis, total RNA was extracted using RNeasy Mini Kit (Qiagen) and treated with DNase I according to the manufacturer's instructions. After assessing the quality of total RNA using an Agilent 2100 Bioanalyzer and RNA Nanochip (Agilent Technologies), 150 ng total RNA was treated to deplete the levels of ribosomal RNA (rRNA) using target-specific oligos combined with rRNA removal beads. Following rRNA removal, mRNA was fragmented and converted into double-stranded cDNA. Adaptor-ligated cDNA was amplified by limit cycle PCR.

After library quality was determined via Agilent 4200 TapeStation and quantified by KAPA qPCR, approximately 60 million paired-end 150 bp sequence reads were generated on the Illumina HiSeq 4000 platform. Quality control and adapter trimming were accomplished using the FastQC (version 0.11.3) and Trim Galore (version 0.4.0) software packages. Trimmed reads were mapped to the Genome Reference Consortium GRCm38 (mm10) murine genome assembly using TopHat2 (version 2.1.0), and feature counts were generated using HTSeq (version 0.6.1). Statistical analysis for differential expression was performed using the DESeq2 package (version 1.16.1) in R, with the default Benjamini-Hochberg *p-value* adjustment method. The Ingenuity Pathway Analysis (IPA) software (QIAGEN), the Gene Set Enrichment Analysis (GSEA) software (UC San Diego, BROAD Ins.), and the R Programming Language software were used to analyze gene signature and pathway enrichment.

NMR analysis of medium

Naïve $CD4^+$ T cells isolated from WT mice were polarized for 72 hrs under T_H17 or iT_{reg} culture conditions. Cells were harvested, washed by PBS, and then re-seeded at a 2×10^6 cells /mL density in a conditional medium (RPMI-1640) containing 4 mM $^{13}C_5$-Glutamine. Medium samples were taken at 0 hr and after 6 hrs incubation and were extracted and lyophilized. The dried samples were reconstituted in deuterated 50 mM phosphate buffer pH 8 containing 8.81 nmole DSS-d_6. NMR spectra were recorded on a Bruker Avance III spectrometer at 16.45 T at 15 °C in a 1.7 mm HCN inverse triple resonance cryoprobe. Presat spectra were recorded with an acquisition time of 2 s with weak irradiation at the HOD frequency during the relaxation delay of 4 s (pulse program: ZGPR). $^1H\{^{13}C\}$ HSQC spectra were recorded with an acquisition time of 0.25 s and a relaxation delay of 1.75 using adiabatic decoupling of ^{13}C (pulse program: hsqcetgpprsisp2.2). The raw data were apodized with a 1 Hz exponential line broadening and linear predicted once (Presat) or a 4-Hz line broadening exponential (HSQC), phased, and baseline corrected using third-order Bernstein polynomials and referenced to the internal DSS at 0 ppm. Peaks were deconvoluted by line fitting using MNOVA and normalized to the DSS resonance at 0 ppm. The HCCH-TOCSY 2D spectrum was acquired by recording the first plane of the 3D experiment in the carbon dimension (pulse program: hcchdigp3d). The acquisition times in the direct and indirect dimensions (f_2 and f_1) are 0.25 s and 0.03 s, respectively. The C-C mixing time was set to 10.9 ms using a DIPSI-3 spin lock scheme with B_1 field strength of 40 kHz. The data were processed with

1 Hz exponential apodization in f_2 and 5 Hz exponential with cosine squared function in the f_1 dimension, and further linear predicted and zero-filled to 8 k and 2k data points, respectively. The spectrum was phased and baseplane corrected before referencing glutamine peaks based on the standard spectrum. Spectral assignments were made by reference to authentic standards of GAB recorded under the same conditions.

Table 1. Cell culture related antibodies, cytokines, and chemicals

InVivoMAb anti-mouse CD3, Bio X cell, Cat# BE0001-1, Clone 145-2C11
InVivoMAb anti-mouse CD28, Bio X cell, Cat# BE0015-1, Clone 37.51
InVivoMAb anti-mouse IL-2, Bio X cell, Cat# BE0043, Clone JES6-1A12
InVivoMAb anti-mouse IL-4, Bio X cell, Cat# BE0045, Clone 11B11
InVivoMAb anti-mouse IFNγ, , Bio X cell, Cat# BE0055, Clone XMG1.2
Recombinant Murine IL-12 p70, PeproTech, Cat# 210-12
Recombinant Human TGF-β1, PeproTech, Cat# 100-21C
Recombinant Murine IL-2, PeproTech, Cat# 212-12
Recombinant Murine IL-7, PeproTech, Cat# 217-17
Recombinant Murine IL-4, PeproTech, Cat# 214-14
Recombinant Murine IL-6, PeproTech, Cat# 216-16
γ-Aminobutyric acid, Sigma, Cat# A2129
FCCP, Sigma, Cat# C2520
Aminoguanidine; AG, Sigma-Aldrich, Cat# A7009
GDH1 Inhibitor, R162 – Calbiochem, Sigma-Aldrich, Cat# 5.38098
Bicuculline, Cayman, Cat# 11727
Picrotoxin, Cayman, Cat# 20771
Flumazenil, Cayman, Cat# 14252
(R,S)-4-Amino-5-hexenoic acid; Vigabatrin, CNH Technologies, Cat# X-1501
Polyketides NV118, Isomerase therapeutics Ltd. Cambridge, Cat# 01-118-s3

Table 2. Cell staining antibodies and dyes

FITC anti-mouse CD4, BioLegend, Cat# 100510, Clone RM4-5
PE/Cyanine7 anti-mouse CD4, BioLegend, Cat# 100422, Clone GK1.5
Pacific Blue™ anti-mouse CD4, BioLegend, Cat# 100428, Clone GK1.5
APC/Cyanine7 anti-mouse CD8a, BioLegend, Cat# 100714, Clone 53-6.7
PE/Cy7 anti-mouse CD69, BioLegend, Cat# 104512, Clone H1.2F3
PE anti-mouse CD25, BioLegend, Cat# 102008, Clone PC61
APC/Cy7 anti-mouse CD90.1 (Thy1.1), BioLegend, Cat# 202520, Clone OX-7
APC anti-mouse CD90.1 (Thy1.1), BioLegend, Cat# 202526, Clone OX-7
PE anti-mouse CD90.2 (Thy1.2), BioLegend, Cat# 105308, Clone 30-H12
APC anti-mouse TCR β chain, BioLegend, Cat# 109211, Clone GL3
PE/Cyanine7 anti-mouse IFN-γ, BioLegend, Cat# 505826, Clone XMG1.2
APC anti-mouse IFN-γ, BioLegend, Cat# 505810, Clone XMG1.2
PE/Cyanine7 anti-mouse IL-17A, BioLegend, Cat# 506922, Clone TC11-18H10.1
APC anti-mouse IL-17A, BioLegend, Cat# 506916, Clone TC11-18H10.1
APC anti-mouse IL-4, BioLegend, Cat# 504105, Clone 11B11
Alexa Fluor® 647 anti-mouse FOXP3, BioLegend, Cat# 126407, Clone MF-14
APC anti-mouse CD62L, BioLegend, Cat# 104412, Clone MEL-14
FITC anti-mouse/human CD44, BioLegend, Cat# 103006, Clone IM7
PerCP anti-mouse CD45.1, BioLegend, Cat# 110726, Clone A20
PerCP anti-mouse CD45.2, BioLegend, Cat# 109826, Clone104
FITC anti-mouse/human/rat ABAT, Santa Cruz Biotechnology, Cat# sc-393769, Clone B-12
APC anti-Hu/Mo ROR gamma (t), eBioscience, Cat# 17-6988-82, Clone AFKJS-9
PE anti-Hu/Mo Phospho-STAT3 (Tyr705), eBioscience, Cat# 12-9033-42, Clone LUVNKLA
PE anti-Hu/Mo Phospho-STAT5 (Tyr694), eBioscience, Cat# 12-9010-42, Clone SRBCZX
Pacific Blue-P-S6Ribosomal Protein (S235/236), Cell Signaling, Cat# 8520S, Clone D57.2.2E
APC anti-BrdU, BioLegend, Cat# 364114
7-AAD Viability Staining Solution, BioLegend, Cat# 420404
Pyronin Y, Sigma-Aldrich, Cat# 92-32-0

Table 3. pMIC-ABAT sequencing

Host-Constructs: pMIC(MSCV-IRES-mCherry)
MCS: EcoRI/Bgl II/SnaBI/BamHI/MfeI/XhoI
Inserts: No tag; include start codon (ATG) and stop codon
PREDICTED: Homo sapiens 4-aminobutyrate aminotransferase (ABAT), transcript variant X2, mRNA
Sequence ID: ref|XM_011522401.1|Length: 5115Number of Matches: 1
Gene-associated gene details
Range 1: 466 to 1333GenBankGraphics Next Match Previous Match
Alignment statistics for match #1

```
Query  91
TCATGGCCTCCATGTTGCTCGCCCAGCGCCTGGCCTGCAGCTTCCAGCACAGCTACCGCC  150
|||||||||||||||||||||||||||||||||||||||||||||||||||||||||||| Subject  466
TCATGGCCTCCATGTTGCTCGCCCAGCGCCTGGCCTGCAGCTTCCAGCACAGCTACCGCC  525

Query  151
TGCTGGTGCCTGGATCCAGACACATTAGTCAAGCTGCAGCCAAAGTCGACGTTGAATTTG  210
|||||||||||||||||||||||||||||||||||||||||||||||||||||||||||| Subject  526
TGCTGGTGCCTGGATCCAGACACATTAGTCAAGCTGCAGCCAAAGTCGACGTTGAATTTG  585

Query  211
ATTATGATGGGCCTCTGATGAAGACGGAAGTCCCAGGGCCTAGATCTCAGGAGTTAATGA  270
|||||||||||||||||||||||||||||||||||||||||||||||||||||||||||| Subject  586
ATTATGATGGGCCTCTGATGAAGACGGAAGTCCCAGGGCCTAGATCTCAGGAGTTAATGA  645

Query  271
AACAGCTGAATATAATTCAGAATGCAGAGGCTGTGCATTTTTTCTGCAATTACGAAGAGA  330
|||||||||||||||||||||||||||||||||||||||||||||||||||||||||||| Subject  646
AACAGCTGAATATAATTCAGAATGCAGAGGCTGTGCATTTTTTCTGCAATTACGAAGAGA  705

Query  331
GCCGAGGCAATTACCTGGTTGATGTGGACGGCAACCGAATGCTGGATCTTTATTCCCAGA  390
|||||||||||||||||||||||||||||||||||||||||||||||||||||||||||| Subject  706
GCCGAGGCAATTACCTGGTTGATGTGGACGGCAACCGAATGCTGGATCTTTATTCCCAGA  765

Query  391
TCTCCTCTGTCCCCATAGGTTACAGCCACCCCGCCCTGCTGAAACTCATCCAACAGCCTC  450
|||||||||||||||||||||||||||||||||||||||||||||||||||||||||||| Subject  766
TCTCCTCTGTCCCCATAGGTTACAGCCACCCCGCCCTGCTGAAACTCATCCAACAGCCTC  825

Query  451
AAAATGCGAGCATGTTTGTCAACAGACCCGCCCTCGGAATCCTGCCTCCGGAGAACTTTG  510
|||||||||||||||||||||||||||||||||||||||||||||||||||||||||||| Subject  826
AAAATGCGAGCATGTTTGTCAACAGACCCGCCCTCGGAATCCTGCCTCCGGAGAACTTTG  885

Query  511
TGGAGAAGCTCCGGCAGTCCTTGCTCTCGGTGGCTCCCAAAGGGATGTCCCAGCTCATCA  570
|||||||||||||||||||||||||||||||||||||||||||||||||||||||||||| Subject  886
TGGAGAAGCTCCGGCAGTCCTTGCTCTCGGTGGCTCCCAAAGGGATGTCCCAGCTCATCA  945

Query  571
CCATGGCCTGCGGCTCCTGCTCCAATGAAAACGCCTTAAAGACCATCTTCATGTGGTACC  630
|||||||||||||||||||||||||||||||||||||||||||||||||||||||||||| Subject  946
CCATGGCCTGCGGCTCCTGCTCCAATGAAAACGCCTTAAAGACCATCTTCATGTGGTACC  1005

Query  631
GGAGCAAGGAAAGAGGGCAGAGGGGCTTCTCCCAGGAGGAGCTGGAGACGTGCATGATTA  690
|||||||||||||||||||||||||||||||||||||||||||||||||||||||||||| Subject  1006
GGAGCAAGGAAAGAGGGCAGAGGGGCTTCTCCCAGGAGGAGCTGGAGACGTGCATGATTA  1065

Query  691
ACCAGGCCCCTGGCTGCCCCGACTACAGCATCCTCTCCTTCATGGGCGCGTTCCATGGGA  750
|||||||||||||||||||||||||||||||||||||||||||||||||||||||||||| Subject  1066
ACCAGGCCCCTGGCTGCCCCGACTACAGCATCCTCTCCTTCATGGGCGCGTTCCATGGGA  1125

Query  751
GGACCATGGGTTGCTTAGCGACCACGCACTCTAAAGCCATTCACAAGATCGACATCCCTT  810
|||||||||||||||||||||||||||||||||||||||||||||||||||||||||||| Subject  1126
GGACCATGGGTTGCTTAGCGACCACGCACTCTAAAGCCATTCACAAGATCGACATCCCTT  1185

Query  811
CCTTTGACTGGCCCATCGCACCGTTCCCACGGCTGAAATACCCTCTGGAAGAGTTTGTGA  870
|||||||||||||||||||||||||||||||||||||||||||||||||||||||||||| Subject  1186
CCTTTGACTGGCCCATCGCACCGTTCCCACGGCTGAAATACCCTCTGGAAGAGTTTGTGA  1245

Query  871
AAGAGAACCAACAGGAGGAGGCCCGCTGTCTGGAAGAGGTGGAGGATCTGATTGTG-AAT  929
|||||||||||||||||||||||||||||||||||||||||||||||||||||||| ||| Subject  1246
AAGAGAACCAACAGGAGGAGGCCCGCTGTCTGGAAGAGGTGGAGGATCTGATTGTGAAAT  1305

Query  930
ATCGGaaaaannn-anGACGGTGGCCGGGATCATCGTGGAG-CCATCCAGTCCNNGG-TG  986
||||||||||    | ||||||||||||||||||||||||| |||||||||||  || || Subject  1306
ATCGGAAAAAGAAGAAGACGGTGGCCGGGATCATCGTGGAGCCCATCCAGTCCGAGGGTG  1365

Query  987
GAGAC-ACCACGCATCCGATGACTTNTTTCgnaa-ctga  1023
||||||||||||||||||| ||||| || |||| Subject  1366
GAGACAACCACGCATCCGATGACTTCTTTCGGAAGCTGA  1404
```

Table 4. Stable isotope tracers

U13C5-Arginine, Cambridge Isotope Laboratories, Inc., Cat# CLM2265
U13C6-Glucose, Cambridge Isotope Laboratories, Inc., Cat# CLM1396
U13C5-Glutamine, Cambridge Isotope Laboratories, Inc., Cat# CLM1822
4-Aminobutyric acid (GABA)(13C4, 97-99%), Cambridge Isotope Laboratories, Inc., Cat# CLM-8666
1,4-BUTANEDIAMINE (PUTRECINE) (13C4, 98%), Cambridge Isotope Laboratories, Inc., Cat# CLM-6574

Table 5. RT-qPCR primers

Gene	primer sequences forward	primer sequences reverse	NCBI Gene ID	Primer Bank ID	Vendor (Sequences of oligonucleotides)
GAD1	AACGTATGATACTTGGTGTGGC	CCAGGCTATTGGTCCTTTGTAAG	Mouse, 14415	145301579c1	Sigma-Aldrich
GAD2	TCCGGCTTTTGGTCCTTCG	ATGCCGCCCGTGAACTTTT	Mouse, 14417	124517708c1	Sigma-Aldrich
ABAT	CTGAACACAATCCAGAATGCAGA	GGTTGTAACCTATGGGCACAG	Mouse, 268860	27370474a1	Sigma-Aldrich
Aldh5a1	CGGTCAAGGAGAGGAGCTTAC	GGACTAGCCCTCGCTTATCTTT	Mouse, 214579	27369748a1	Sigma-Aldrich
Akr7a5	CGGCCAGTCCGAGAACATC	TTCAGTGACTTCCCTTCCCAG	Mouse, 110198	27659728a1	Sigma-Aldrich
Glyr1	GAAACTGGGCCGGTATCCTC	GGTAAGGCTTTAGTTGTTCCACT	Mouse, 74022	21312000a1	Sigma-Aldrich
Slc52a2	GGGCTCCTACCTGATGACACT	GCACAGGACCCATGAGAGTA	Mouse, 52710	21313230a1	Sigma-Aldrich
Slc6a1	GAAAGCTGTCTGATTCTGAGGTG	AGCAAACGATGATGGAGTCCC	Mouse, 232333	30520131a1	Sigma-Aldrich
Slc6a11	TGTTGAGCGTAGCTGGAGAGA	AGCAGATGAAAAACACCACGTA	Mouse, 243616	27370360a1	Sigma-Aldrich
Slc6a12	GGTCCCTGAGGAAGGAGAGAT	GGGGATGAAGAAAGCTCCACC	Mouse, 14411	19526806a1	Sigma-Aldrich
Slc6a13	CAGTACACCAACCAGGGAGG	GCCAGGACAACGATGTAGTAGA	Mouse, 14412	21362295a1	Sigma-Aldrich
Slc32a1	ACCTCCGTGTCCAACAAGTC	CAAAGTCGAGATCGTCGCAGT	Mouse, 22348	6678569a1	Sigma-Aldrich
Gabra1	AAAAGTCGGGGTCTCTCTGAC	CAGTCGGTCCAAAATTCTTGTGA	Mouse, 14394	6753936a1	Sigma-Aldrich
Gabra2	GGACCCAGTCAGGTTGGTG	TCCTGGTCTAAGCCGATTATCAT	Mouse, 14395	6679901a1	Sigma-Aldrich
Gabra3	ATGTGGCACTTTTATGTGACCA	CCCCAGGTTCTTGTCGTCTTG	Mouse, 14396	31560695a1	Sigma-Aldrich
Gabra4	ACAATGAGACTCACCATAAGTGC	GGCCTTTGGTCCAGGTGTAG	Mouse, 14397	33468895a1	Sigma-Aldrich
Gabra5	TGACCCAAACCCTCCTTGTCT	GTGATGTTGTCATTGGTCTCGT	Mouse, 110886	30578386a1	Sigma-Aldrich
Gabra6	TGCCCAAGCTCAACTTGAAGA	GCCGTAGACGGTTGTCATAGC	Mouse, 14399	6679905a1	Sigma-Aldrich
Gabarap	AAGAGGAGCATCCGTTCGAGA	GCTTTGGGGGCTTTTTCCAC	Mouse, 56486	9789961a1	Sigma-Aldrich
Gabarapl1	GGACCACCCCTTCGAGTATC	CCTCTTATCCAGATCAGGGACC	Mouse, 57436	10181206a1	Sigma-Aldrich
Gabarapl2	TCGGGCTCTCAGATTGTTGAC	ATGGCCTTCTCGGAGGGAA	Mouse, 93739	31542873a1	Sigma-Aldrich
jakmip1 (gababrbp)	ACCGCCTACATCTCGGAACT	GCAGCTCACCTTCCTTGATCTT	Mouse, 76071	30409980a1	Sigma-Aldrich
Gabrb1	TCCCGTGATGGTTGCTATGG	CCGCAAGCGAATGTCATATCC	Mouse, 14400	6679907a1	Sigma-Aldrich
Gabrb2	ATGTCGCTGGTTAAAGAGACG	CTGCCACTCGGTTGTCCAAA	Mouse, 14401	6679909a1	Sigma-Aldrich
Gabrb3	CTGCTGCCAATCTGGCTTTC	CGTAGCCTTTCAACAGCTTGTC	Mouse, 14402	26350247a1	Sigma-Aldrich
Gabrd	ATTGGGGACTACGTGGGCT	CCACATTCACAGGAGCACC	Mouse, 14403	6679913a1	Sigma-Aldrich
Gabre	CCTTCAGTGGAGGGTTGGAC	ATTCAGGCGGAGTTAGAGGCT	Mouse, 14404	8393396a1	Sigma-Aldrich
Gabrg1	TGTGGAGTCAAACTAGAGGAGTG	TTCCCAGATGCAGGGTTAGTA	Mouse, 14405	26332763a1	Sigma-Aldrich
Gabrg2	ATGAGTTCGCCAAATACATGGAG	GGAGCAGAATCCACAGCGT	Mouse, 14406	28916679a1	Sigma-Aldrich
Gabrg3	GACAGTCGCCTTCGATTCAAC	AGCCTCCGCTGTTTTAGAATTT	Mouse, 14407	6679917a1	Sigma-Aldrich
Gabrp	CAGACCCACGGCTAGTGTTC	AGAGGCGGATGAGCCTGTT	Mouse, 216643	20379971a1	Sigma-Aldrich
Gabrq	ATGGGCATCCGAGGTATGCT	ATCCAGAATGACTTCAGGCGT	Mouse, 57249	10048422a1	Sigma-Aldrich
Gabrr1	CGAGGAGCACACGACGATG	GTGAAGTCCATGTCAACCTCTG	Mouse, 14408	6679919a1	Sigma-Aldrich
Gabrr2	ATGCCTTATTTGATGAGACTCGC	CCACACCTACAGGGATGGC	Mouse, 14409	6679921a1	Sigma-Aldrich

Gabrr3	CACCCTAAACGTGAACAACTGT	TCCAATAGTGCCTGAGGTAAAAC	Mouse, 328699	124487130c2	Sigma-Aldrich
Gabbr1	GCACAGGACACAATGAAAACAG	AGCAAATGTACTCGACTCCCA	Mouse, 54393	5051395a1	Sigma-Aldrich
Gabbr2	AAGACCCCATAGAGGACATCAA	GGGTGGTACGTGTCTGTGG	Mouse, 242425	29611612a1	Sigma-Aldrich
DBI	GAATTTGACAAAGCCGCTGAG	CCCACAGTAGCTTGTTTGAAGTG	Mouse, 13167	6681137a1	Sigma-Aldrich
Arg1	CTCCAAGCCAAAGTCCTTAGAG	AGGAGCTGTCATTAGGGACATC	Mouse, 11846	158966684c1	Sigma-Aldrich
Arg2	TCCTCCACGGGCAAATTCC	GCTGGACCATATTCCACTCCTA	Mouse, 11847	6753110a1	Sigma-Aldrich
ODC1	GGTTCCAGAGGCCAAACA	CAGCGTGCCATCATCCT	Mouse, 111148	N/A	Sigma-Aldrich
Aldh1a1	ATACTTGTCGGATTTAGGAGGCT	GGGCCTATCTTCCAAATGAACA	Mouse, 11668	7304881a1	Sigma-Aldrich
DAO	GGTGGCAAGAGGAGTGGATG	TGGGATGATGTACGGAGAGTTG	Mouse, 13142	15929683a1	Sigma-Aldrich
Aldh4a1 (PDH)	CGATGGAAGCACACCTCTTCT	GGCGACAACTGGTACTGTATATC	Mouse, 212647	34328415a1	Sigma-Aldrich
Tubulin (beta-2 microglobulin)	TTCTGGTGCTTGTCTCACTGA	CAGTATGTTCGGCTTCCCATTC	Mouse, 12010	144227219c1	Sigma-Aldrich

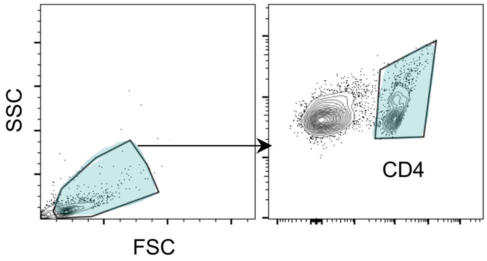

Supplemental Figure 1. For analysis of CD4+ T cell proliferation, infiltrating and intracellular cytokines (Fig 4c, Fig 4g, Fig 4i, Fig 4k, Fig 5f, Fig 5h, Extended Data Fig 2b, Fig 4a-d, Fig 5, Fig 6b, Fig 8b, Fig 9c, and Fig 10): FSC-SSC-H gating was used as preliminary gating for lymphocyte population followed by analysis of $CD4^+$ T cells, then checked the intracellular cytokines expression.

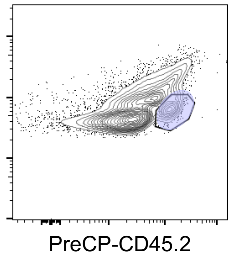

Supplemental Figure 2. For *in vivo* adaptive transfer experiment, gating strategy for flow cytometry analysis was preliminarily performed by gating for CD45.2 stain marker (Extended Data Fig 6a, OVA antigen-specific).
