# Supplementary material for: GAB functions as a bioenergetic and signalling gatekeeper to control T cell inflammation
Source: Nat Metab. 2022 Oct 3;4(10):1322–35. doi: 10.1038/s42255-022-00638-1 (PMC9584824; doi:10.1038/s42255-022-00638-1)
Supplement: Supplementary file 2 — Reporting Summary [file 42255_2022_638_MOESM2_ESM.pdf]

nature portfolio

Corresponding author(s): Ruoning Wang

Last updated by author(s): 2022/08/03

Reporting Summary

Nature Portfolio wishes to improve the reproducibility of the work that we publish. This form provides structure for consistency and transparency in reporting. For further information on Nature Portfolio policies, see our Editorial Policies and the Editorial Policy Checklist.

Statistics

For all statistical analyses, confirm that the following items are present in the figure legend, table legend, main text, or Methods section.

n/a	Confirmed	
☐	☒	The exact sample size (n) for each experimental group/condition, given as a discrete number and unit of measurement
☐	☒	A statement on whether measurements were taken from distinct samples or whether the same sample was measured repeatedly
☐	☒	The statistical test(s) used AND whether they are one- or two-sided<br>*Only common tests should be described solely by name; describe more complex techniques in the Methods section.*
☒	☐	A description of all covariates tested
☒	☐	A description of any assumptions or corrections, such as tests of normality and adjustment for multiple comparisons
☐	☒	A full description of the statistical parameters including central tendency (e.g. means) or other basic estimates (e.g. regression coefficient) AND variation (e.g. standard deviation) or associated estimates of uncertainty (e.g. confidence intervals)
☐	☒	For null hypothesis testing, the test statistic (e.g. F, t, r) with confidence intervals, effect sizes, degrees of freedom and P value noted<br>*Give P values as exact values whenever suitable.*
☒	☐	For Bayesian analysis, information on the choice of priors and Markov chain Monte Carlo settings
☒	☐	For hierarchical and complex designs, identification of the appropriate level for tests and full reporting of outcomes
☒	☐	Estimates of effect sizes (e.g. Cohen's d, Pearson's r), indicating how they were calculated

Our web collection on statistics for biologists contains articles on many of the points above.

Software and code

Policy information about availability of computer code

Data collection	Flow cytometry by using Novocyte (ACEA Biosciences) software (version 2000); quantitative PCR by using BIO-RAD CFX284TM Real-Time PCR Detection System; RNA-seq quality control and adapter trimming were accomplished using the FastQC (version 0.11.3) and Trim Galore (version 0.4.0) software packages, trimmed reads were mapped to the Genome Reference Consortium GRCm38 (mm10) murine genome assembly using TopHat2 (version 2.1.0), and feature counts were generated using HTSeq (version 0.6.1); 13C-tracer data by using Gas Chromatography-Mass Spectrometry (GC-MS) as standard method; medium metabolites by using Liquid Chromatography-Mass Spectrometry (LC-MS) (metabolon), NMR, or bioanalyzer (YSI, version 2900) as standard method; Oxygen consumption rate (OCR) by using seahorse XFe96 Analyzer (Agilent Technologies)
Data analysis	Flow cytomertric analysis were performed with FlowJo software (TreeStar, version 10.6); RNAseq analysis were performed using the DESeq2 package (version 1.16.1) in R, with the default Benjamini-Hochberg p-value adjustment method, the Ingenuity Pathway Analysis (IPA) software (QIAGEN, version 01-20-04), the Gene Set Enrichment Analysis (GSEA) software (UC San Diego, BROAD Ins. version 4.1.0), and the R Programming Language software (version 4.2.1); Oxygen consumption rate (OCR) were analysis by using the Seahorse Wave Software (Seahorse, Agilent Technologies. version 2.6); Statistical data analysis and generation of graphs by using GraphPad Prism (version 8.0.1).

For manuscripts utilizing custom algorithms or software that are central to the research but not yet described in published literature, software must be made available to editors and reviewers. We strongly encourage code deposition in a community repository (e.g. GitHub). See the Nature Portfolio guidelines for submitting code & software for further information.

Data

Policy information about availability of data

All manuscripts must include a data availability statement. This statement should provide the following information, where applicable:

- Accession codes, unique identifiers, or web links for publicly available datasets
- A description of any restrictions on data availability
- For clinical datasets or third party data, please ensure that the statement adheres to our policy

Raw RNA-seq datasets generated for this study can be found in the GEO accession GSE190818 (reserved released data at 08/02/2022). https://www.ncbi.nlm.nih.gov/geo/query/acc.cgi. The authors declare that all other data (including the Metabolon) and materials supporting the findings of this study are available within the article (and supplementary/ extended information files). Original LC-MS metabolomics data and code please address correspondence and requests for materials to Ruoning Wang (ruoning.wang@nationwidechildrens.org).

Field-specific reporting

Please select the one below that is the best fit for your research. If you are not sure, read the appropriate sections before making your selection.

☒ Life sciences ☐ Behavioural & social sciences ☐ Ecological, evolutionary & environmental sciences

For a reference copy of the document with all sections, see nature.com/documents/nr-reporting-summary-flat.pdf

Life sciences study design

All studies must disclose on these points even when the disclosure is negative.

Sample size	Sample sizes determined on the basis of previous experience in previous experiments. For Metabolon, LC-MS, GS-MS studies 3 independent samples for determination (referring Ratnikov, B. et al. Bioinfornatics 2006; Bunk, B. et al. Bioinfornatics 2006; Evans, A.M. et al. Anal Chem 2009); for EAE studies, 3-5 independent experiments with mice based on the experimental design (referring Wu, R. et al. Sci Adv 2020; Chen, X. et al. Sci Imm 2022); for Elisa, YSI, qPCR measurements, 3-6 independent samples were used; for in vivo adoptive transferred experiment, 3-5 independent mice were used (referring Wu, R. et al. Sci Adv 2020; Chen, X. et al. Sci Imm 2022); for cell culture related experiments such as FACS, 3 independent samples at least were performed. The number of independent experiments (at least 3) is a standard sample size to accurately detect differences in cell biology filed (referring multiple publications in Nature, Nature Metabolism, Nature Immunology, et al).
Data exclusions	No data exclusion
Replication	All experiments were conducted with at least two independent experiments and multiple biological replicates (except metabolon was performed one time and 3 biological replicates), and the details was provided in corresponding figure legends.
Randomization	All studies were performed on age and gender matched animals. Animals were randomized prior to experiments.
Blinding	Experiments were not performed blinded because all analysis were performed using same gating as control under the same condition.

Reporting for specific materials, systems and methods

We require information from authors about some types of materials, experimental systems and methods used in many studies. Here, indicate whether each material, system or method listed is relevant to your study. If you are not sure if a list item applies to your research, read the appropriate section before selecting a response.

Materials & experimental systems

n/a	Involved in the study	
☐	☒	Antibodies
☒	☐	Eukaryotic cell lines
☒	☐	Palaeontology and archaeology
☐	☒	Animals and other organisms
☒	☐	Human research participants
☒	☐	Clinical data
☒	☐	Dual use research of concern

Methods

n/a	Involved in the study	
☒	☐	ChIP-seq
☐	☒	Flow cytometry
☒	☐	MRI-based neuroimaging

Antibodies

Antibodies used	For cell culture: InVivoMAb anti-mouse CD3 (clone 145-2C11, Bio X Cell, Cat# BE0001-1)<br>InVivoMAb anti-mouse CD28 (clone 37.51, Bio X Cell, Cat# BE0015-1)<br>InVivoMAb anti-mouse IL-2 (clone JES6-1A12, Bio X Cell, Cat# BE0043)

InVivoMAb anti-mouse IL-4 (clone 11B11, Bio X Cell, Cat# BE0045)
InVivoMAb anti-mouse IFN gamma (clone XMG1.2, Bio X Cell, Cat# BE0055)
For flow cytometry: Anti-mouse CD4-FITC (clone RM4-5, Biolegend, Cat# 100510, dilution 1:100)
anti-mouse CD4-PE/Cyanine7 (clone GK1.5, Biolegend, Cat# 100422, dilution 1:100)
anti-mouse CD4-Pacific Blue (clone GK1.5, Biolegend, Cat# 100428, dilution 1:100)
anti-mouse CD8-APC/Cyanine7 (clone 53-6.7, Biolegend, Cat# 100714, dilution 1:100)
anti-mouse CD62L-APC (clone MEL-14, Biolegend, Cat# 104412, dilution 1:100)
anti-mouse CD44-FITC (clone IM7, Biolegend, Cat# 103006, dilution 1:100)
anti-mouse CD69-PE/Cy7 (clone H1.2F3, Biolegend, Cat# 104512, dilution 1:100)
anti-mouse CD25-PE (clone PC61, Biolegend, Cat# 102008, dilution 1:100)
anti-mouse CD45.1-PerCP (clone A20, Biolegend, Cat# 110726, dilution 1:100)
anti-mouse CD45.2-PerCP (clone 104, Biolegend, Cat# 109826, dilution 1:100)
anti-mouse Thy1.1-APC/Cy7 (clone OX-7, Biolegend, Cat# 202520, dilution 1:100)
anti-mouse Thy1.1-APC (clone OX-7, Biolegend, Cat# 202526, dilution 1:100)
anti-mouse Thy1.2-PE (clone 30-H12, Biolegend, Cat# 105308, dilution 1:100)
anti-mouse TCR beta-APC (clone GL3, Biolegend, Cat# 109211, dilution 1:100)
anti-mouse IFN gamma-APC (clone XMG1.2, Biolegend, Cat# 505810, dilution 1:100)
anti-mouse IFN gamma-PE/Cyanine7 (clone XMG1.2, Biolegend, Cat# 505826, dilution 1:100)
anti-mouse IL-17A-APC (clone TC11-18H10.1, Biolegend, Cat# 506916, dilution 1:100)
anti-mouse IL-17A-PE/Cyanine7 (clone TC11-18H10.1, Biolegend, Cat# 506922, dilution 1:100)
anti-mouse IL-4-APC (clone 11B11, Biolegend, Cat# 504105, dilution 1:50)
anti-mouse/human/rat ABAT-FITC or purified (clone B-12, Santa Cruz Biotechnology, Cat# sc-393769, dilution 1:50)
anti-mouse FoxP3-Alexa Fluor® 647 (clone MF-14, Biolegend, Cat# 126407, dilution 1:100)
anti-Hu/Mo ROR gamma (t) (clone AFKJS-9, eBioscience, Cat# 17-6988-82, dilution 1:50)
anti-Hu/Mo Phospho STAT3 (Tyr705) (clone LUVNKLA, eBioscience, Cat# 12-9033-42, dilution 1:50)
anti-Hu/Mo Phospho STAT5 (Tyr694) (clone SRBCZX, eBioscience, Cat# 12-9010-42, dilution 1:50)
P-S6Ribosomal Protein-Pacific Blue (S235/236) (clone D57.2.2E, Cell Signaling, Cat# 8520S, dilution 1:50)
anti-Alexa Fluro 647 BrdU (Biolegend, Cat# 364114, dilution 1:100)
Pyronin Y (Sigma-Aldrich, Cat# 92-32-0, dilution 1:100)

Validation

Reactivity of above antibodies are commercially available and validated for indicated applications, all information on manufacturer's homepage:
https://bxcell.com/
https://www.biolegend.com/
https://www.scbt.com/home
https://www.thermofisher.com/us/en/home/life-science/antibodies/ebioscience
https://www.cellsignal.com/
https://www.sigmaaldrich.com/US/en

Animals and other organisms

Policy information about studies involving animals; ARRIVE guidelines recommended for reporting animal research

Laboratory animals

C57BL/6 (WT), Flippase (B6.129S4Gt(ROSA)26Sortm1(FLP1)Dym/RainJ), OT-II (B6.Cg-Tg(TcraTcrb)425Cbn/J), CD45.1+ (B6.SJL-PtprcaPepcb/BoyJ), Rag1-/- (B6.129S7-Rag1tm1Mom/J), IL17A-IRES-GFP-KI (C57BL/6-Il17atm1Bcgen/J), FoxP3GFP+ (C57BL/6-Tg(Foxp3-GFP)90Pkraj/J), and Gabrb3fl (B6;129-Gabrb3tm2.1Geh/J) mice were obtained from the Jackson Laboratory (JAX, Bar Harbor, ME). Mice with one targeted allele of ABAT on the C57BL/6 background (ABATtm1a(EUCOMM)Hmgu) were generated by The European Conditional Mouse Mutagenesis Program (EUCOMM). The mice were first crossed with a transgenic Flippase strain (B6.129S4Gt(ROSA)26Sortm1(FLP1)Dym/RainJ) to remove the LacZ-reporter allele and then crossed with the CD4-Cre strain to generate T cell-specific ABAT knockout strain (ABAT cKO). OT-II mice were crossed with CD4Cre ABAT cKO mice to generate the OT- II CD4Cre ABAT cKO mice. OT-II mice were crossed with Thy1.1+ mice (B6.PL-Thy1a/CyJ) to generate the OT-II Thy1.1 mice. Gabrb3fl mice were crossed with the CD4-Cre strain to generate T cell-specific Gabrb3 knockout strain (Gabrb3 cKO). Both male and female mice, with age-matched (6-12 weeks old) were used in the experiments. Mice were housed under controlled conditions: rodent housing rooms are kept at 73 degree Fahrenheit, with alarms set at 69 and 78 degrees, 30–70% relative humidity, and 12:12 light-dark cycle. Food and water was available for all animals. Low Fat diet were provided (Envigo 2920, the irradiated form of 2020X*). Mice were maintained and euthanized (by carbon dioxide asphyxiation followed by cervical dislocation) under protocols approved by the Institutional Animal Care and Use Committee of the Research Institute at Nationwide Children's Hospital (IACUC; protocol number AR13-00055). *https://insights.envigo.com/hubfs/resources/data-sheets/2020x-datasheet-0915.pdf

Wild animals

This study does not include any wild animal.

Field-collected samples

This study does not include samples collected from the field.

Ethics oversight

Animal protocols were approved by the Institutional Animal Care and Use Committee of the Research Institute at Nationwide Children's Hospital.

Note that full information on the approval of the study protocol must also be provided in the manuscript.

Flow Cytometry

Plots

Confirm that:

☒ The axis labels state the marker and fluorochrome used (e.g. CD4-FITC).

☒ The axis scales are clearly visible. Include numbers along axes only for bottom left plot of group (a 'group' is an analysis of identical markers).

☒ All plots are contour plots with outliers or pseudocolor plots.

☒ A numerical value for number of cells or percentage (with statistics) is provided.

Methodology

Sample preparation	For development analysis, spleen, lymph nodes and thymus were passed through 70 micron filters; for EAE infiltrating T cell analysis, the CNS (brain and spinal cord), spleen, and peripheral lymph nodes were collected and mashed to make the single-cell solution. The cell suspension was centrifuged on a 30%/70% Percoll gradient at 500 g for 30 min to isolate mononuclear cells from the CNS.<br>All cells were stained in PBS containing 2% (w/v) BSA and the appropriate antibodies from Biolegend. For analyzing intracellular cytokine IFN-gamma and IL-17A, T cells were stimulated for 4 hrs with eBioscience™ Cell Stimulation Cocktail (eBioscience) before being stained with cell-surface antibodies. Cells were then fixed and permeabilized using FoxP3 Fixation/ Permeabilization solution according to the manufacturer's instructions (eBioscience). Cell proliferation was assessed by CFSE staining per the manufacturer's instructions (Invitrogen). Cell viability was evaluated by 7AAD staining per the manufacturer's instructions (Biolegend). For analyzing DNA/RNA content, cells were collected and stained with surface markers before being fixed with 4% paraformaldehyde for 30 min at 4°C, followed by a step of permeabilization with FoxP3 permeabilization solution (eBioscience). Cells were stained with 7AAD for 5 min and then stained with pyronin-Y for 30 min before being analyzed by flow cytometer with PerCP channel for 7AAD (DNA) and PE channel for pyronin-Y (RNA). Protein synthesis assay kit (Item No.601100, Cayman) was used for analyzing protein content. Briefly, cells were incubated with O-propargyl-puromycin (OPP) for 1 hr, then were fixed and stained with 5 FAM-Azide staining solutions before being analyzed by flow cytometer with FITC channel. For analyzing cell cycle profile, cells were incubated with 10 µg/mL BrdU for 1 hr, followed by cell surface staining, fixation, and permeabilization according to Phase-Flow Alexa Fluro 647 BrdU Kit (Biolegend).
Instrument	Novocyte
Software	FlowJo version 10.6
Cell population abundance	Bulk cell isolation including naive CD4 T cells and naive tTreg cells were performed using kits (MojoSort, BioLegend) individually, ensuring a purity of >90%-95% defined as the ratio of target cells and total cells. For RNAseq, WT and ABAT cKO T cells were activated and harvested at 36 hrs separately, used for RNA extraction (around 100-500 ng/ul/sample). For Metabolon assay, differentiated CD4 subsets such as TH1, TH17 and iTreg for 72 hrs, FACS determined to ensure a purity of about 70% defined as the ratio of IFN gamma (TH1), IL17A (TH17) and FoxP3 (iTreg) and total cells. For in vitro Treg cell suppression assays, differentiated iTreg for 72 hrs, FACS determined to ensure a purity of about 70% FoxP3 (iTreg) cells, co-cultured with Tconv cells in indicated ratio as described in Methods. Brifely, purity of cell fractions were determined by flow cytometry ensuring a appropriate purity based on the experiment purpose. No cell sorting were performed at this time.
Gating strategy	Gating strategies are shown respectively: For analysis of alive cells (Fig 2j, Fig 5b, Fig 5e, Fig S3a, Fig S3e, Extended Data Fig 9a, and Fig 9b): PerCP-7AAD gating for alive cells (Fig 2j upper) followed by analysis of GFP-surface expressed IL17A, or detected CFSE cell proliferation, or CD25/CD69 cell activation marker; for analysis of CD4+ T cell proliferation, infiltrating and intracellular cytokines (Fig 4c, Fig 4g, Fig 4i, Fig 4k, Fig 5f, Fig 5h, Extended Data Fig 2b, Fig 4a-d, Fig 5, Fig 6b, Fig 8b, Fig 9c, and Fig 10): FSC-SSC-H gating was used as preliminary gating for lymphocyte population followed by analysis of CD4+ T cells, then checked the intracellular cytokines expression (gating strategy in Supplementary Information); for in vivo adaptive transfer experiment, gating strategy for flow cytometry analysis was preliminarily performed by gating for CD45.2 stain marker (Extended Data Fig 6a, OVA antigen-specific), by gating for TCR-beta (Extended Data Fig 6d, homeostatic), and then checked the cell ratio, proliferation or cytokines expression; for Treg cell suppression assay, gating strategy for flow cytometry analysis was preliminarily performed by gating for CD45.2 stain marker to distinguish the Tconv cells, then separated WT cells and KO cells by Thy1.1 marker and Thy1.2 marker (Fig 6e), and then checked the proliferation.

☒ Tick this box to confirm that a figure exemplifying the gating strategy is provided in the Supplementary Information.
