# Supplementary figures and images for: GAB functions as a bioenergetic and signalling gatekeeper to control T cell inflammation
Source: Nat Metab. 2022 Oct 3;4(10):1322–35. doi: 10.1038/s42255-022-00638-1 (PMC9584824; doi:10.1038/s42255-022-00638-1)

(refer main Fig 2a)

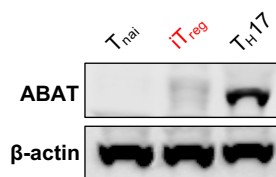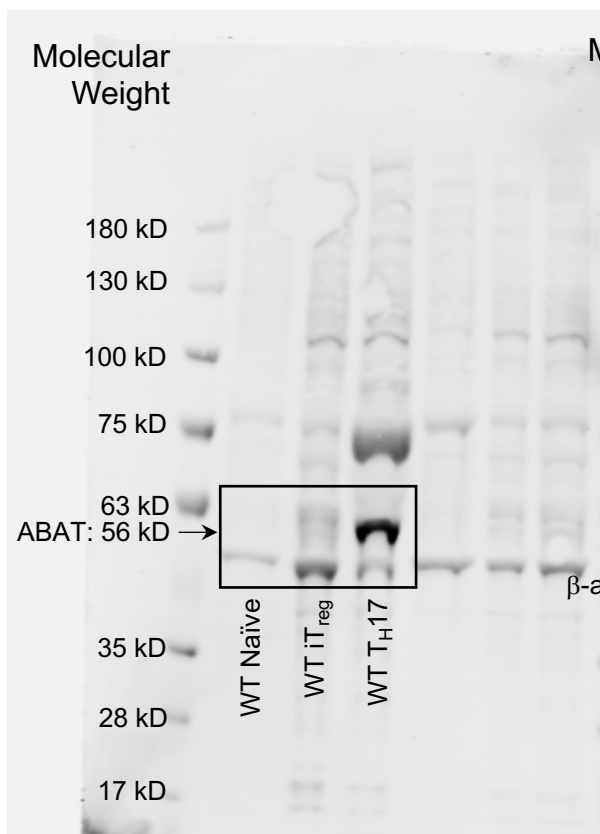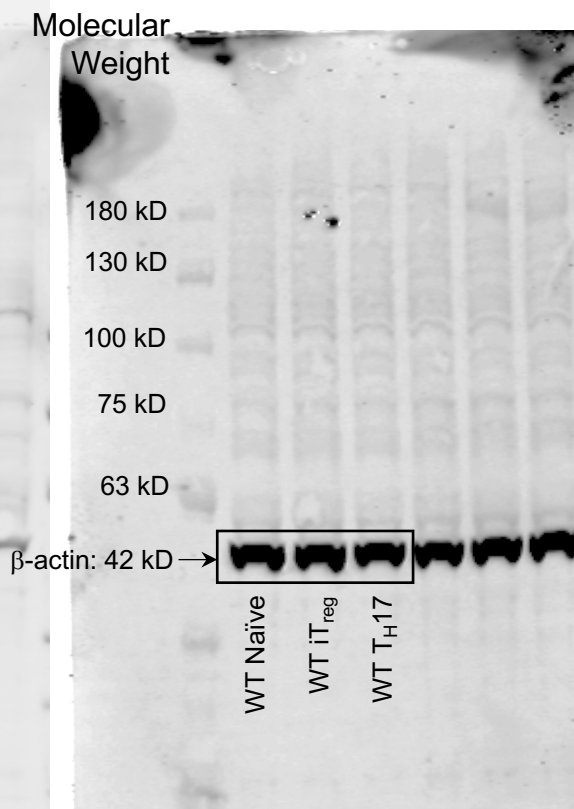

(refer **main Fig 4a**)

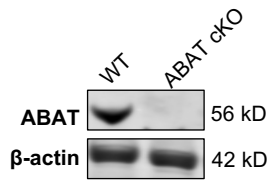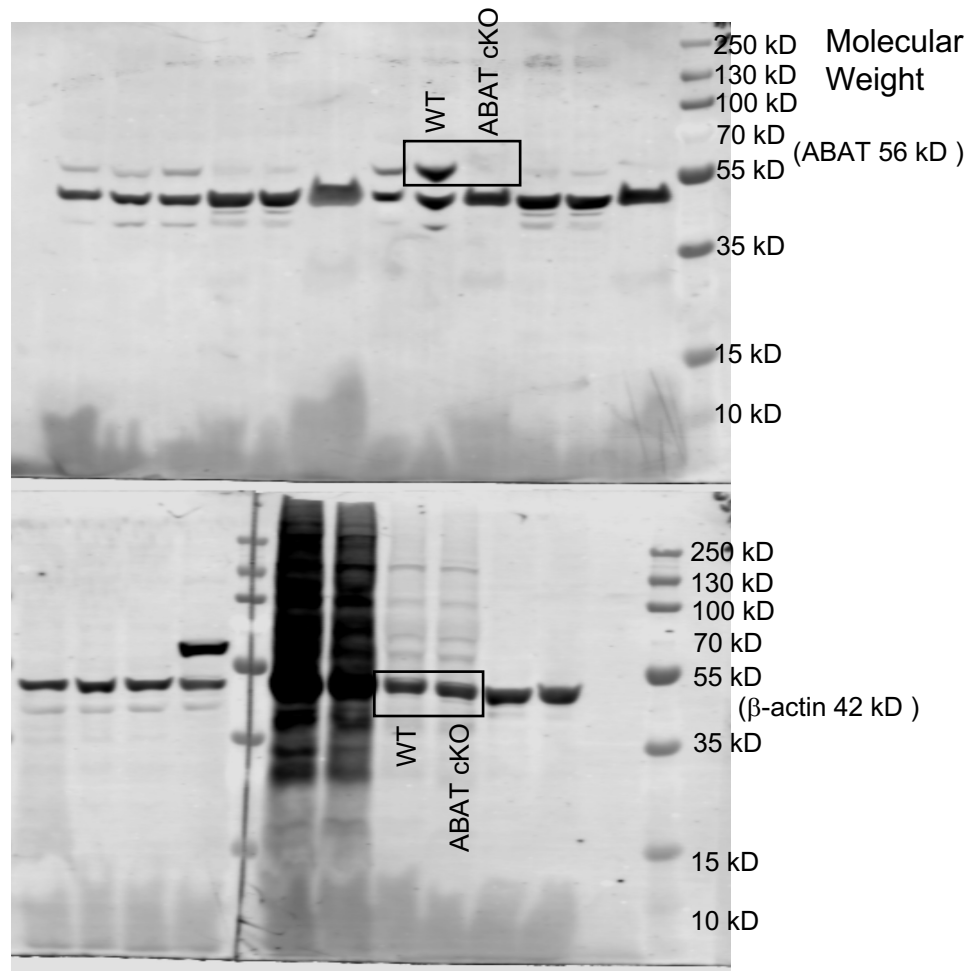

Supplement: Source Data Fig. 2 — Unprocessed Western blots for Fig. 2a. [file 42255_2022_638_MOESM4_ESM.pdf]
